# Reciprocal exchanges across multilayered networks show an emerging patron–client system led by salaried households

**DOI:** 10.1017/ehs.2026.10061

**Published:** 2026-07-03

**Authors:** Joon Hwang, Neil G. MacLaren, David Nolin, Siobhán Cully, Nurul Alam, Mary K. Shenk

**Affiliations:** 1 Department of Anthropology, Pennsylvania State University, University Park, Pennsylvania, USA; 2 Department of Mathematics, State University of New York at Buffalo, Buffalo, New York, USA; 3 Independent researcher; 4 Department of Anthropology, Rutgers University–New Brunswick, New Brunswick, New Jersey, USA; 5 International Centre for Diarrhoeal Disease Research, Dhaka, Bangladesh

**Keywords:** cooperation, market integration, social inequality, social network, Bangladesh

## Abstract

Evolutionary models of social inequality suggest that status differentiation can emerge within cooperative networks through sustained asymmetric exchanges between high- and low-value resources, giving rise to patron–client relationships. From a network perspective, such asymmetric relationships take the form of multilayered reciprocity, where different types of resources are exchanged across domains. By analysing multilayered support networks in Matlab, Bangladesh, a region undergoing rapid market integration, we examine how multilayered reciprocity is differently leveraged depending on socioeconomic status and how these exchange patterns reflect an emerging patron–client system in an increasingly market-integrated community. Evidence of multilayered reciprocity is found for salaried households who provide cash and receive household items and/or labour services, but similar patterns are not found for landowners or local political leaders. Further, observed asymmetries in exchange patterns suggest that salaried households may be emerging as new patrons providing sought-after resources (especially cash) to clients with limited access to such resources, replacing a traditional patron–client system based on land ownership and political leadership. Our findings highlight a mechanism by which human cooperation can give rise to new forms of social inequality, offering a framework for extending evolutionary models to explain cooperation and inequality in changing socioeconomic contexts.

## Graphical abstract

**Figure:**
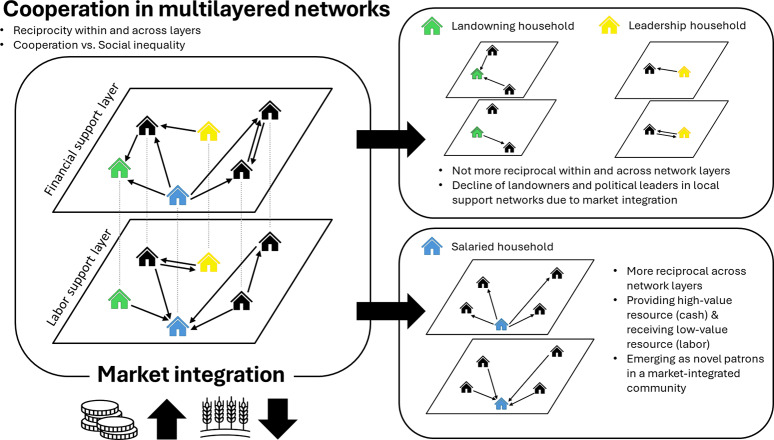

## Social media summary

Salaried households trade cash for labor and goods, emerging as new patrons amid market integration in rural Bangladesh.

## 1. Introduction

Reciprocity plays an important role in the evolution of cooperation as it enables the sharing of risks across individuals in unpredictable environments, where successful individuals provide surpluses to those in need and receive help when they are in turn unsuccessful (Cashdan, 1985; Winterhalder et al., 1999). Studies on human sharing have often focused on how reciprocal exchanges reduce risk within a single behavioural domain, particularly in the context of food-sharing (Alvard & Nolin, 2024; Gurven et al., 2000; Hawkes et al., 2001; Kaplan et al., 1985). While in-kind support is an important form of human risk-buffering, less is understood regarding how interdependence arises through trading of different types of resources (Demps & Winterhalder, 2019; Jaeggi et al., 2016; Winterhalder, 1996). Drawing on early work on cross-cutting social ties (Gluckman, 1955), anthropologists have long recognized the multiplex (multilayered) nature of social relationships, where individuals are linked through overlapping and multi-stranded social ties that cut across multiple domains of social life (Barnes, 1954; Kapferer, 1969; Mitchell, 1973). In multilayered social networks, sharing patterns that seem unidirectional in a single domain may turn out to be reciprocal across domains, as two or more different types of goods and services are exchanged between individuals (Atkisson et al., 2020; Baggio et al., 2016). For example, patterns of multilayered reciprocity have been observed among Ache foragers whose food provisioning was reciprocated with childcare and mating opportunities (Kaplan et al., 1985), and among Tsimané horticulturalists who exchanged labour for childcare and sick care, and horticultural produce for hunted meat (Jaeggi et al., 2016). Analyses confined to a single domain therefore risk mischaracterizing interdependent relationships by obscuring the redundancy and coordination generated through overlapping social ties (Mitchell, 1973).

### 1.1. *Patron*–*client relationships and the evolution of social inequality in multilayered networks*

Reciprocal exchanges across domains can foster interdependent relationships based on a division of labour (Jaeggi et al., 2016), but they also have the potential to develop into systems of social inequality known as ‘patron–client relationships’, where access to high-value resources, such as land in agricultural societies (Scott, 1972; Wolf, 1966) or livestock in pastoral societies (Moritz et al., 2011; Pelican, 2012; Sneath, 1993), is exchanged for labour, political support, or other lower-value resources that are more evenly distributed within the community. While patron–client relationships arise in contexts of material inequality, they are sustained through asymmetric but reciprocal exchanges that actively transform unequal access to resources into durable relations of dependence and authority. In rural southern Italy and Spain, for example, wealthy local notables selectively intervened on behalf of poorer villagers in disputes with tax collectors, police, or courts. Such assistance was neither legally required nor uniformly available but was extended in expectation of public loyalty, political alignment, and continued support, resulting in relations characterized as ‘lopsided friendship’, reflecting reciprocity across domains rather than mere differences in material wealth (Wolf, 1966).

Recently, evolutionary researchers have begun to recognize such patron–client relationships as key mechanisms in the emergence of social inequality (Mattison et al., 2016; Redhead & Power, 2022; von Rueden, 2023). Rather than viewing hierarchy solely as a product of coercion or dominance, new evolutionary models emphasize how mutually beneficial, yet asymmetric exchanges of resources can lead to durable hierarchies, especially when cooperation is organized within network structures (Redhead & Power, 2022). From this perspective, inequality evolves not despite cooperation but through it, as adaptive reciprocity becomes structured by persistent disparities in resource control (Mattison et al., 2016).

Multilayered social networks offer a useful framework for identifying how asymmetric cooperation can remain mutually beneficial, particularly when individuals vary in access to and demand for distinct types of resources. Previous studies have shown that individuals with greater material wealth were *less* engaged in reciprocal support relationships in Mpimbwe, Tanzania (Kasper & Borgerhoff Mulder, 2015) and in Lamalera, Indonesia (Nolin, 2010). This may occur when wealthy individuals are less reliant on within-domain reciprocity (Franzen & Eaves, 2007; Gurven et al., 2015), because they are equipped with alternative risk-reduction strategies such as cash income or storable foods. Yet among these wealthy individuals, the demand for (or marginal value of) other types of resources, such as political support or prestige that cannot be readily purchased with wealth, can be greater than the demand for (or marginal value of) material resources (Demps & Winterhalder, 2019; Winterhalder, 1996). Such differences in demand (or marginal utility) suggest that in multilayered networks where actors vary in their comparative advantages, cross-domain reciprocity could be leveraged to generate interdependent yet asymmetric relationships; indeed, such relationships may have been evolutionarily favoured for sustaining cooperation amid unequal access to valued resources (Mattison et al., 2016; Pisor & Gurven, 2018).

### 1.2. Market integration and the transformation of sharing networks

The emergence of patron–client relationships reflects not only individual variation in the quantity of resources but also broader ecological contexts that determine which kinds of resources confer patron status. For example, patron–client relationships have been commonly described in contexts of differential agricultural land ownership where landowners may serve as patrons and those who work on their land as clients (Scott, 1972; Wolf, 1966), or in pastoral contexts where patrons may be the owners of large herds (Moritz et al., 2011; Pelican, 2012; Sneath, 1993).

Such relationships are often thought to disappear, or at least become less important, as communities undergo market integration, the process of socioeconomic transition from subsistence-based to market-oriented activities (BurnSilver et al., 2016; Lu, 2007; Mattison et al., 2022; Shenk et al., 2016). If resource sharing is strongly motivated and maintained by the desire to reduce unpredictable variability in subsistence outcomes, the availability of alternative means for risk reduction, such as storage of food or wealth, could result in the decline of sharing practices (Behrens, 1992; Bohannan, 1959). Cash income generated from wage labour provides another means for storing wealth to buffer risk, dramatically reducing people’s need to rely on reciprocal sharing practices, which are risky because of their vulnerability to free riders (Franzen & Eaves, 2007). In Shipibo, Peru, for instance, increased cash cropping and selling rice to the market eventually led to a decline in traditional sharing of faunal foods acquired by hunters and fishers, as they could no longer expect that their sharing would be reciprocated by the rice-producing neighbours (Behrens, 1992). It is thus possible that as sharing networks erode, the interdependencies that sustain patron–client relationships may also weaken, as fewer individuals are positioned to offer support that others cannot readily secure through the market (Scott & Kerkvliet, 1973; Weingrod & Morin, 1971).

However, a growing number of studies suggest that the erosion of traditional sharing practices and egalitarianism is not an inevitable outcome of market integration. Among Tsimané horticulturalists in Bolivia, for example, market integration did not completely replace traditional sharing networks, and wealthier individuals are more actively involved in sharing networks, both in terms of the number of partners and the amount they give to those partners (Gurven et al., 2015). In two Iñupiat communities, those who were highly engaged in market activities were also disproportionately involved in subsistence activities, sharing, and cooperation (BurnSilver et al., 2016). The persistence of sharing practices in market-integrated communities may be explained by reputational and relational benefits associated with sharing. Through sharing resources with others, the sharers signal generosity and commitment to the group interests, which in turn enhances social status (Ready & Power, 2018) and fosters long-term partnerships (Jaeggi et al., 2016). With these additional benefits alongside the potential for simple risk reduction, sharing networks can persist in a market-integrated community, and their maintenance may crucially rely upon the provision of the social climbers who try to hold a higher rank or the high-status individuals who struggle to remain at the top of a local hierarchy. Once again, these reputational and relational returns are generated through multiplex social ties, where acts of sharing simultaneously operate between economic, social, and status-related domains (Mitchell, 1973).

### 1.3. Reconfiguration of patron–client relationships in market-integrated communities

Even when sharing practices persist, the dynamics of patron–client relationships may shift as the broader economic and ecological contexts surrounding cooperation are transformed by market integration (Mattison et al., 2016; von Rueden, 2023). From an evolutionary perspective, biological market theory (Noë & Hammerstein, 1994, 1995) suggests that cooperation is strongly shaped by strategic partner choice, where individuals preferentially associate with those who possess advantageous traits, such as greater resources or social influence, and thus can potentially offer the greatest benefits (Barclay & Willer, 2007). In this view, patrons are not simply those with more resources but those whose particular advantages, whether land, livestock, wage income, or political ties, make them desirable exchange partners in a given environmental context.

In rapidly transitioning communities where the market exchange system is firmly established but access to formal credit and insurance systems remains limited, cash may become a critical resource that elevates individuals as desirable cooperative partners. Among the Tsimané of Bolivia, growing market integration has enabled some individuals to use cash income from wage labour or cash cropping to hire fellow villagers for field labour (von Rueden, 2023). If cash, the distribution of which is highly asymmetric, continues to be exchanged for labour, which is more evenly distributed within a community, power imbalances may accumulate from such asymmetric exchanges, resulting in novel patron–client relationships in market-integrated communities (Caudell et al., 2015; Rashid, 2012). At the same time, individuals who once held influence through land ownership or livestock wealth may lose their standing as patrons, as their traditional forms of capital become less relevant in market-oriented economies.

Thus, to investigate how patron–client relationships are reconfigured in market-integrated communities, it is essential to examine the domains of support that are most critical for survival, in this case, access to cash, and the socioeconomic statuses that are likely to confer access to such resources, such as salaried jobs. At the same time, it remains important to include more traditional domains of support, such as food and labour, and traditional forms of socioeconomic capital, such as land ownership and local political leadership, in order to compare how different forms of capital and exchange relate to cooperation and inequality under changing economic conditions.

### 1.4. Research questions: multilayered support networks and patron–client relationships in Matlab

To this end, we analysed demographic, economic, and network data collected across three adjacent *baris* (neighbourhoods) in Matlab, Bangladesh, a rural area that is rapidly undergoing market integration, with growing access to wage labour, salaried jobs, and consumer markets alongside more traditional agricultural livelihoods. We constructed a multilayered network based on household-level exchanges across three support domains: financial support (money), material support (food or household goods), and labour support (childcare, agricultural labour, and other physical help). Our multilayered network consists of nodes (vertices), defined as the social units among which relationships are specified (Moreno, 1934), which in this study correspond to individual respondent households. These households vary in socioeconomic status, allowing us to examine whether social network patterns are contingent on the land ownership, salaried income, and/or local political leadership status of the households. Building on this framework, we ask three interrelated research questions to explore how cooperation and social inequality are structured in the context of market integration:

**1. How are support relationships structured within and across financial, material, and labour support domains of the multilayered networks in the context of market integration?** In Matlab, access to cash is limited but increasingly central to livelihoods, raising the possibility that financial support may play a distinct role in structuring exchanges relative to other forms of support. Empirically, such a shift would be reflected in greater number of connections and stronger within-layer reciprocity in financial support, as well as more frequent cross-domain exchanges linking financial support to material or labour support. Examining how reciprocity operates within and between support domains can reveal whether financial support is becoming more prominent relative to other forms of exchange in a market-integrating setting.

**2. Do emerging forms of socioeconomic status (e.g., salaried income) predict different patterns of reciprocity within and across network layers, compared to more traditional forms of status (e.g., land ownership, political leadership), in a market-integrated community?** Salaried income offers households a predictable flow of cash, which has become increasingly important for daily life in Matlab. Thus, salaried households may be emerging as key actors in facilitating exchange relationships. On the other hand, more traditional forms of socioeconomic status, such as land ownership and political leadership, have historically conferred influence, but their relevance for structuring support relationships may be changing as livelihoods shift towards market-oriented activities. These differences can be assessed by comparing whether salaried households, relative to landowners and political leaders, maintain a larger number of support ties, occupy more central or influential positions within specific support layers, and engage more frequently in reciprocal exchanges within and across domains.

**3. Do patterns of cross-domain reciprocity among households reflect asymmetric exchanges consistent with patron**–**client dynamics, particularly in relation to emerging and traditional forms of socioeconomic status?** In patron–client relationships, patrons provide scarce, high-value resources to clients, and clients reciprocate with more abundant, lower-value resources, creating an asymmetric interdependence. In network terms, such dynamics would be evident in the direction of cross-domain exchanges, with households occupying patron positions more likely to provide financial support in return for labour or material assistance, rather than receiving cash in exchange for these forms of support. Examining the directionality of cross-domain exchanges in relation to household socioeconomic status allows us to assess whether emerging or traditional forms of status are more closely associated with patron–client relationships in Matlab.

## 2. Methods

### 2.1. Study context

Demographic, economic, and network data of 331 individuals from 79 households in 3 geographically adjacent *baris* were collected within a village in Matlab between November 2017 and January 2018. Respondent households were identified through complete enumeration of the entire population residing within the study area so that we did not miss any household that may play a role in shaping community-wide support networks. Located 3 km west of the nearest market town and facing the Dhonagoda River, a branch of the Meghna River, these baris are representative of rural communities shifting away from subsistence activities and towards more market-oriented ones (Mattison et al., 2022). Focus group data, incorporated briefly in the discussion, were also collected in Matlab in 2018 with individuals residing in both the town of Matlab Bazaar and in periurban and rural areas of Matlab.

Matlab residents traditionally practiced agriculture and fishing (Razzaque & Streatfield, 2002), but these have declined as primary occupations in response to increasing market integration. Market integration has gradually altered various aspects of life in Matlab over the last few decades, as villagers increasingly rely on cash and are rapidly adopting newly emerging occupations including wage labour, small businesses, and, for a few, education-based jobs with fixed salaries (Novak, 1993; Shenk et al., 2016; Siddiqui, 2016; Sznajder et al., 2021). While about 30% of the resident households own small- to medium-sized farmlands, and it is not rare to observe villagers working in the fields, most adult men consider their primary occupations as wage labourers, salaried workers, or business owners (tea stalls near the village or small shops in the bazar) rather than as farmers. Many households have one or more family members who are currently working abroad and households with return migrants are often more affluent than others due to wealth accumulated during labour out-migration. Market integration has also impacted social relationships among Matlab villagers, as moneylending between individuals has become a crucial part of social exchange (Alam et al., 2019; Hoque et al., 2015; Jackson & Young, 2016; Mirelman et al., 2019; Rashid, 2012), alongside more traditional types of social networks for sharing of material items and labour assistance.

This study was approved by the Ethical Review Committee of International Center for Diarrheal Disease Research, Bangladesh (icddr,b) and the Institutional Review Board at Pennsylvania State University. Informed consent was obtained from all participants, and all aspects of the study were performed in accordance with guidelines and regulations provided by icddr,b and Pennsylvania State University.

### 2.2. Demographic and socioeconomic attributes of households

Social and demographic features of the households used in the analysis include kinship, differences in material wealth, and geographic distances. A binary indicator of kinship between two households is employed in the analysis to investigate whether kinship ties affect the tie formation in social support networks. Household-level material wealth is measured by aggregating the market values of housing materials and other household assets (SI ‘Household material wealth survey’). Household wealth reflects liquid and material assets relevant to short-term support exchanges and excludes land ownership, which is analysed separately as a distinct and less readily mobilized form of socioeconomic status. GPS coordinates were recorded at the front entrance for each household, and the distances between the households were calculated using ArcGIS Pro.

We also examine three forms of socioeconomic status that may influence the emergence of patron–client relationships in Matlab: land ownership, salaried income, and local political leadership. Land ownership traditionally has been considered high-status because landowners were more financially and nutritionally secure, often employing others as sharecroppers or agricultural labourers. Land ownership was measured through self-report data at the household level and coded as binary (Islam et al., 2020; Phillips et al., 1988; Stewart et al., 1990). In contrast, salaried income is emerging as a new form of social and economic capital, as it affords individuals financial stability with direct access to cash. Thus, a binary indicator of whether the household head earns a salaried income is used for analysis to distinguish households with stable cash income from those without. Finally, some individuals occupy leadership positions in local politics, which may increase the motivations of network members for cooperation. Local political leadership was measured by asking the respondents whether any household members have a leadership position, such as a bari head, village head, or member of a local governmental organization; this variable was also coded as binary. Descriptive statistics of household demographic and socioeconomic attributes are presented in SI Tables 1 and 2. SI Table 1 summarizes the characteristics of all households included in the study, while SI Table 2 compares these characteristics by land ownership, salaried income, and political leadership status.

### 2.3. Social support networks

Multilayered networks composed of three support layers are drawn from a survey conducted with the adult female members of the village, one from each household (*N* = 79). Social ties were elicited using name generators, a standard survey method in which respondents are asked to identify individuals with whom they maintain specified types of social relationships (Marsden, 1990; Power, 2017). In this study, respondents answered six name-generator questions covering different forms of social support (see SI, ‘Social support network survey’). In double-sampled financial support questions, they named individuals from whom household members would borrow money and to whom they would lend money in case of emergency. The material support layer is also constructed based on double-sampled questions where respondents listed individuals from whom household members would borrow food or household items and to whom they would lend these items. Finally, the labour support layer was created by asking villagers to name individuals whom they would ask for help if female household members needed help caring for their children or grandchildren and if male household members needed help with any of their work in the house or community. We structured these three support layers into multilayered support networks, as the set of nodes is the same across all layers and the nodes are connected to different sets of nodes on each layer while maintaining their identities across layers (Atkisson et al., 2020).

### 2.4. Data analysis

As a preliminary and descriptive analysis, we use a set of node-level network measures to characterize heterogeneity in household network position across support domains. These measures summarize different aspects of connectivity and structural position, providing an overview of how households are situated within each support network prior to formal modelling. Degree centrality summarizes the number of support ties a household provides (in-degree for support-receiving ties, out-degree for support-providing ties), reflecting direct participation in exchange relationships. Eigenvector centrality captures whether households are connected to others who are themselves well connected (Newman, 2008), highlighting embeddedness within dense regions of the network. Betweenness centrality reflects the extent to which households connect otherwise unconnected households (Borgatti, 2005; Freeman et al., 1979), indicating potential brokerage roles. Finally, we apply the Hyperlink-Induced Topic Search algorithm (Kleinberg et al., 1999), which assigns hub and authority scores based on the recursive structure of directed ties, distinguishing households that primarily direct support towards others who themselves receive support from many sources (hub), from those that primarily receive support from households that direct support to many others (authority). In combination, these measures allow us to evaluate whether emerging and traditional forms of socioeconomic status are associated with differences in who provides versus receives support (degree centrality), occupying more embedded (eigenvector centrality) or bridging (betweenness centrality) positions within each support layer, and whether households disproportionately provide support to others who experience persistent or recurrent needs and therefore receive support from many sources (hub), or receive support from households that routinely act as major providers of support within local networks (authority), patterns that are indicative of asymmetric exchange relationships central to patron–client dynamics.

Next, we use motif analysis to test whether these three types of status have similar or different effects on reciprocity within and across domains. In network analysis, motifs refer to recurring and significant patterns of interconnections between network actors observed within or across network layers (Milo et al., 2002) such as reciprocity within dyads (e.g., two individuals connected mutually; Felmlee et al., 2021) or transitivity within triads (e.g., two connected individuals having a mutual acquaintance; Holland & Leinhardt, 1971; Shizuka & McDonald, 2015). Attention to such small relational configurations has a long history in sociology and social psychology, where dyads, triads, and cycles were treated as fundamental building blocks of social structure and cohesion (Cartwright & Harary, 1956; Davis, 1967; Holland & Leinhardt, 1971). Building on this tradition, Milo et al. (2002) formalized motif analysis as a statistical framework by comparing the frequency of these local configurations in empirical networks to those observed in randomly generated networks, allowing researchers to identify patterns that occur more often than expected by chance.

**Figure 1.**
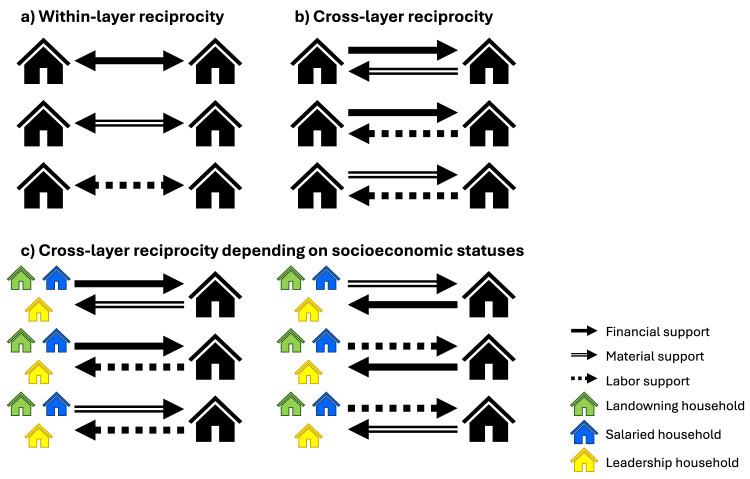
Reciprocity (a) within and (b) between layers and (c) possible patterns of between-layer reciprocity depending on socioeconomic statuses. 1 long description.

We extend classical motif analysis in single-layer networks to examine complex arrangements of ties between status-labelled nodes (households with different types of status) within and across network layers (Bianconi, 2018). In other words, our status-labelled motif analysis accommodates the possibility that households may not only have reciprocal ties within and across layers (Fig. 1a,b) but may engage in these differently based on their socioeconomic status (Fig. 1c). In cases where complex network structures (i.e., multilayered networks) and/or complex network patterns (i.e., status-dependent reciprocity) are difficult to incorporate into standard statistical models, motif analysis can be useful due to its flexibility to accommodate almost any type of network motif.

For such comparison to be valid, however, these randomly simulated networks should be capable of reproducing some fundamental attributes of the empirical networks (Artzy-Randrup et al., 2004). In other words, random networks in the comparison group should realistically represent the general principles regarding how social relationships are formed in the study area. Thus, we build what is called a ‘fundamental model’ using the multilayered Exponential Random Graph Model (ERGM) that defines how social relationships are formed within and across multiple network layers. The multilayered ERGM is useful in constructing such a fundamental model for our analysis, not only because it estimates how household characteristics are associated with the log-odds of tie formation between pairs of households (Hunter et al., 2008) but also because it can do so when the households have ties with others across multiple network layers, accounting for multilayered network structures (Krivitsky et al., 2020). By incorporating multilayered ERGMs into motif analysis, the effects of socioeconomic status on within- and cross-layer reciprocity can be differentiated from the effects of other constraints, allowing us to rigorously explore interdependence and social inequality under multilayered network structures.

The following variables are employed in the fundamental model to constrain tie formation and thereby help the model produce a more realistic landscape of social relationships between the households: (a) Geographic and social constraints include physical distance, kinship, and wealth differences between households. (b) Basic features of the empirical network include the number of edges (ties), reciprocal dyads, transitive triads (SI Fig. 1, left), and isolates (SI Fig. 1, right) within each layer, which allow our simulated networks to closely align with the structural characteristics of the empirical network, improving the goodness-of-fit of the fundamental model. Finally, (c) the multiplex nature of the network is captured using both same direction ties and reciprocal ties across layers. Same direction ties occur when one household receives support from or provides support to the other household in two different layers. If one household receives support from another household in one layer and provides support to that household in another layer, such relationships are counted as cross-layer reciprocal ties. In specifying the fundamental model, we adopted a parsimonious set of structural and dyadic controls, which achieved good overall goodness-of-fit across network layers while avoiding instability or degeneracy in model estimation. A detailed discussion of the rationale for selecting and evaluating candidate control variables, including the iterative process used to assess model fit and stability, is provided in the SI (‘Multilayered Exponential Random Graph Model’).

We then simulate 1,000 random networks where tie formation between nodes is constrained by the fundamental model and compare status-labelled reciprocity motifs between the empirical and simulated networks to see whether and how network patterns vary between households depending on focal socioeconomic status variables. It should be noted here that our fundamental model conditions on geographic distance, kinship, and household wealth but does not include salaried income, land ownership, and political leadership. As a result, simulated networks are random with respect to the variables of interest, while still reflecting the baseline principles that shape social relationships in the study area and reproducing key structural features of the empirical network on average. By comparing the observed number of ties within these groups to their simulated equivalents, we can determine whether the parameters of the fundamental model are sufficient to explain the observed network outcomes (empirical motif values within the range of simulated motif values), or whether instead salaried income, land ownership, and political leadership affect social relationships beyond what the fundamental model predicts (empirical motif values outside the range of simulated motif values).

## 3. Results

### 3.1. Visualization and descriptive statistics of multilayered support networks

**Figure 2.**
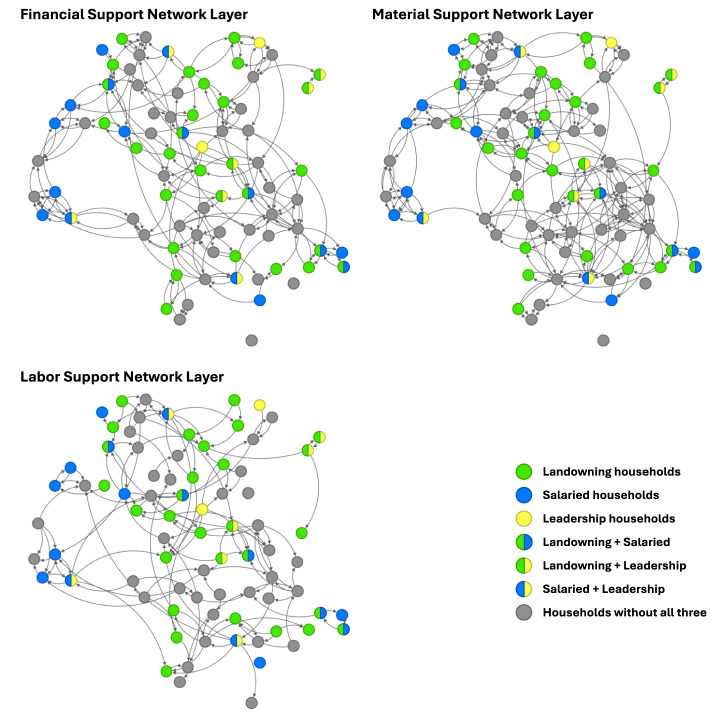
Visualization of multilayered support networks among 79 households in Matlab. Figure 2 long description.

Figure 2 visualizes the multilayered support network among Matlab households (*n* = 79) as a directed graph composed of a common set of nodes and multiple sets of ties (edges) corresponding to distinct support domains. In this representation, nodes denote households, and directed edges represent reported support relationships from the provider to the recipient. Separate network layers capture financial support (money), material support (food or household goods), and labour support (childcare, agricultural labour, and other physical assistance), allowing households to be connected by different types of directed ties across domains.

Νotes: Each node represents a household, and arrows between two nodes indicate support relationships between them. Nodes in green represent land-owning households, nodes in blue represent salaried households, and nodes in yellow represent households whose members have political leadership positions. Nodes having multiple colours indicate that the households have multiple types of status. Nodes in grey represent households not having any of the three types of socioeconomic status.

Network density indicates that the labour support layer (=0.022) is more sparsely connected than the other two layers (financial = 0.029, material = 0.039), indicating that Matlab households have fewer connections to their neighbours through labour support (=1.73) than in the other two layers (financial = 2.29, material = 3.04). We also examine how network characteristics vary depending on land ownership, salaried income, and political leadership status of the households (SI Table 3). Salaried households have more support-providing ties (out-degree) than non-salaried households in financial and material support network layers (Wilcoxon rank-sum test; *W* = 274.5, *p* < 0.01 for financial support, *W* = 323, *p* = 0.024 for material support), while leadership households have more support-providing ties in terms of labour support (*W* = 134.5, *p* < 0.01). Salaried households show higher eigenvector centrality in the financial support layer and higher betweenness centrality in the labour support layer, but only with marginal statistical significance (*W* = 356.5, *p* = 0.07). We also find significantly higher hub scores for salaried households in the financial support layer (*W* = 269, *p* < 0.01) and among leadership households in the labour support layer (*W* = 143, *p* < 0.01), indicating that these households support those who get support from many others. In contrast, no significant differences in network characteristics are observed between landowning and landless households. These comparisons are intended as descriptive diagnostics of node-level network position across socioeconomic groups; conditional effects of household attributes on tie formation are examined in the ERGM and motif analyses that follow.

### 3.2. Fundamental model: the effects of network structures, household characteristics, and within- and cross-layer reciprocity

We construct the fundamental model by fitting a multilayered ERGM (Krivitsky et al., 2020) to our network data, calculating how household characteristics and reciprocity within and across layers contribute to the formation of multilayered support networks in Matlab (SI Table 5). The fundamental model is also constrained by the structural features of the empirical networks to improve goodness-of-fit (SI ‘Multilayered Exponential Random Graph Model’). Geographic distance and kinship between households are significant predictors of network ties, as the odds of a tie are 18% lower with each additional 100 m of distance (SI Table 5, ‘Distance’) and 21% higher between the households related through kinship (SI Table 5, ‘Kinship’). Differences in material wealth between households have domain-specific effects on tie formation, as a 1,000 USD difference in household wealth increases the odds of receiving financial and material support from the wealthier household by 17% and 13%, respectively, but the effect of wealth differences is not significant in the labour support layer (SI Table 5, ‘Wealth difference’).

Reciprocity within support layers (Fig. 1a) has significant effects on tie formation in multilayered networks (SI Table 5, ‘Reciprocity’). The odds that a Matlab household lends money to a household from which it borrows money are approximately 12 times higher than in the absence of such a reciprocal tie and vice versa. Receiving material support from a particular household similarly results in 132 times increased odds of supporting that household in the same layer. The effect of reciprocity in the labour support layer, however, is significantly negative, with the odds of reciprocating labour support reduced by 77%.

We further find that reciprocity works not only within a single layer but also between the layers of different support types (SI Table 5, ‘Cross-layer reciprocity’). Two households have reciprocal ties across layers when one of them provides one type of support while the other household provides one or more different types of support (Fig. 1b). A household that receives financial support from another household has 3.8 times higher odds of providing labour support to the same household and vice versa. Likewise, having an incoming/outgoing tie in the material support layer increases the odds of having an outgoing/incoming tie in the labour support layer by 6.7 times. In contrast, the effect of reciprocity between financial and material support networks is not significant.

### 3.3. Motif analysis: the effects of socioeconomic statuses on reciprocity within and across layers

In order to assess the effect of socioeconomic status on reciprocity within and between network layers, we compared the frequency of status-labelled motifs in the empirical network to the frequencies of status-labelled motifs in a sample of synthetic networks generated from our fundamental model. If the values of a specific motif (e.g., reciprocity) are similar between empirical and random networks, we cannot rule out the possibility that a given network pattern is random with regard to socioeconomic status of the households. In contrast, significant differences in motif values between the empirical and simulated/randomized networks will allow us to infer that salaried income, land ownership, and/or political leadership have significant effects on shaping the network patterns of the households.

**Figure 3.**
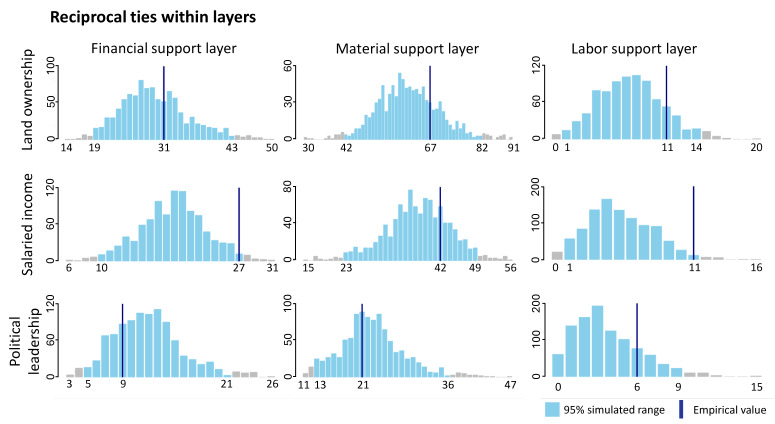
Comparison of within-layer reciprocal ties between empirical networks (navy lines) and simulated networks (bars). Figure 3 long description.

*Notes*: Light blue bars show 95% range of simulated values. The numbers on the *x*-axis indicate the minimum and maximum of the simulated values, lower and upper limits of the 95% range, and the empirical values.

**Figure 4.**
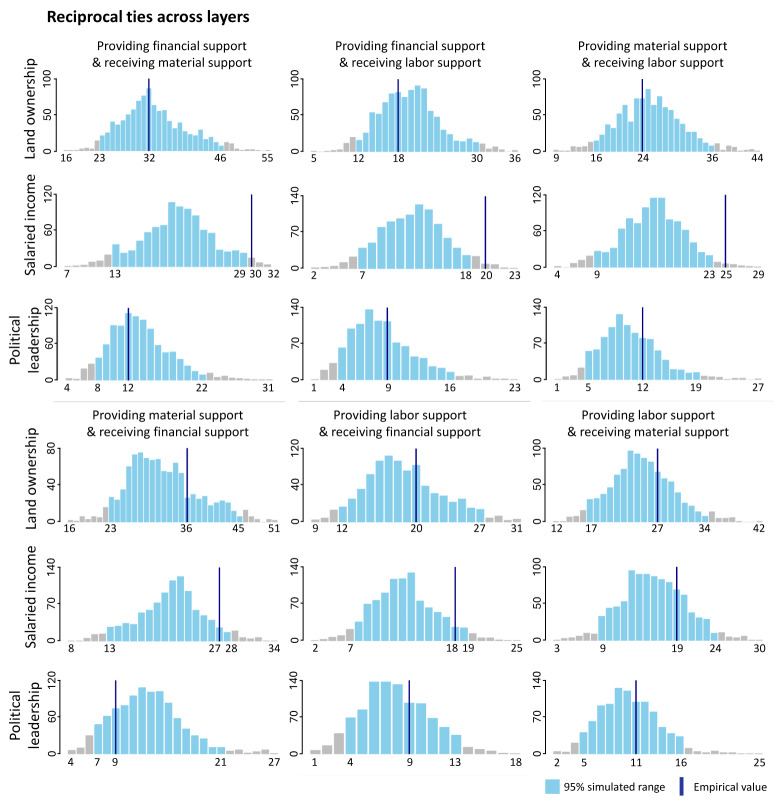
Comparison of cross-layer reciprocal ties between empirical networks (navy lines) and simulated networks (light blue bars). Figure 4 long description.

*Notes*: Light blue bars show the 95% range of simulated values. The numbers on the *x*-axis indicate the minimum and maximum of the simulated values, lower and upper limits of the 95% range, and the empirical values.

The results from the motif analysis are graphically presented in Figs. 3 and 4. Bar plots in the panels of Fig. 3 compare the number of within-layer reciprocal ties in the empirical networks (navy lines) to those observed in the simulated networks, especially to their 95% ranges (light blue bars), and how they vary depending on socioeconomic statuses (rows) and support layers (columns). For example, landowning households have 31 reciprocal ties in the financial support layer of the empirical networks. This value nears the mean of the same motif observed in the simulated networks (95% of the simulations are between 19 and 43), indicating that reciprocity of the landowning households within the financial support layer is unsurprising given our fundamental model. We find that land ownership, salaried income, and political leadership do not particularly influence reciprocity within layers, as the empirical values of the within-layer reciprocal ties are within the 95% range of simulated values for all types of socioeconomic status in all three layers (Fig. 3). However, we find that salaried households tend to have more reciprocal relationships in the empirical layers of the financial and labour support networks than they do in the randomly simulated layers, as the empirical values border the upper limits of the 95% ranges (Monte Carlo [MC] *p*-value = 0.062 [financial support], 0.054 [labour support]) suggesting that salaried income has marginally significant effects on forming reciprocal ties within the financial and labour support layers.

We also examine the number of cross-layer reciprocal ties between empirical networks and simulated networks and the results are visualized in Fig. 4, which is identical to Fig. 3 except for the fact that six different forms of exchanges can be observed across layers (Fig. 1c). Here, we find that only salaried households actively exchange one type of resources for another, specifically by exchanging cash for household items (30 ties), cash for labour (20 ties), and household items for labour (25 ties) (Fig. 4, top three). These empirical values exceed the upper limits (29, 18, and 23 ties, respectively) of the 95% range of the simulated values (MC *p*-value = 0.022), indicating that salaried households have greater numbers of cross-layer reciprocal ties beyond what can be explained by the fundamental model. In contrast, salaried households are not particularly more likely to exchange household items for cash, labour for cash, or labour for household items than what is expected under the fundamental model, as the empirical values are all within the 95% simulated ranges (Fig. 4, bottom three). Household land ownership and political leadership do not have influence on any of the six forms of cross-layer reciprocity examined.

## 4. Discussion

Human cooperation is often sustained through reciprocity, which enables individuals to buffer risk by mutual support over time (Cashdan, 1985). While most studies have focused on reciprocity within a single food-sharing domain (Hawkes et al., 2001; Kaplan et al., 1985), cooperation often spans multiple behavioural domains (Atkisson et al., 2020). In these multilayered networks, individuals may trade different kinds of resources, depending on their relative access to certain resources and the types of support they need most (Demps & Winterhalder, 2019). Such cross-domain reciprocity highlights the multilayered nature of human cooperation (Atkisson et al., 2020; Baggio et al., 2016) by which seemingly unidirectional support within a single domain can be reciprocal across domains. When reciprocal exchanges are distributed across domains, analyses that focus on only one type of exchange risk classifying coordinated systems of support as unreciprocated or altruistic, producing false negatives in assessments of cooperation.

This insight echoes early anthropological arguments that multiplex social ties are fundamental to social cohesion, as they coordinate obligations and expectations across different arenas of social life (Barnes, 1954; Boissevain, 1973; Kapferer, 1969; Mitchell, 1973). From this perspective, multiplexity is not merely a descriptive feature of social networks but a mechanism through which coordination is achieved, allowing individuals to align expectations and sustain interdependence without requiring balance within any single exchange domain. This insight helps reconcile anthropological observations of dense, multi-stranded social relations with formal models of cooperation that emphasize coordination and expectation alignment as central to human sociality (Barrett & Skyrms, 2017; Skyrms, 2001).

Such cross-layer exchanges may be both mutually beneficial and also asymmetric when one party consistently provides scarcer or more valued resources in return for more abundant ones, giving rise to durable hierarchies including patron–client relationships (Scott, 1972; Wolf, 1966). Recent evolutionary perspectives emphasize that social inequality may emerge not in opposition to cooperation but through it (Mattison et al., 2016; Redhead & Power, 2022), as persistent disparities in valued resources shape partner choice and asymmetric interdependence. Our findings contribute to this view by showing how cross-domain reciprocity in a market-integrated community both facilitates cooperation while simultaneously reinforcing social inequality.

### 4.1. Within- and cross-layer reciprocity in a market-integrated community

Our results from the multilayered ERGM provide evidence that social exchanges create reciprocal relationships not only within a single domain but also across different domains. While borrowing money from another household strongly increases the odds of lending money to that household, it also increases the odds of providing labour as well. A similar pattern is observed between material and labour support domains, as material support tends to be reciprocated through both material and labour support. Interestingly, within-domain reciprocity is not observed for labour support and in fact the provision of labour support decreases the odds of receiving labour support from the same household. The absence of reciprocity within the labour support domain, however, does not mean that the principle of reciprocity does not operate, because our results also show that labour support is reciprocated through financial and material support. Thus, observed cross-domain reciprocity highlights the multilayered nature of human cooperation (Atkisson et al., 2020; Baggio et al., 2016) by which seemingly unidirectional support within a single domain can be reciprocal across domains.

These results suggest that Matlab villagers prefer labour-for-cash and labour-for-tool exchanges over in-kind exchanges of labour for labour. Such patterns of cross-domain reciprocity make sense under the context of market integration where people are increasingly reliant on cash for their livelihoods (Caudell et al., 2015; Fafchamps & Lund, 2003). In Matlab, cash is in high demand for purchasing food, paying bills, and educating children, but not every villager has equal access to it due to limited employment opportunities for salaried jobs (Jackson & Young, 2016; Rashid, 2012). Thus, some households facing cash shortages may borrow money from affluent neighbours, but it may not always be possible for them to pay back the borrowed money in the form of cash (Kabeer, 2001; Mirelman et al., 2019). In such cases, one alternative way of reciprocating is to provide childcare and/or physical labour to the lending households. This explanation is, however, not applicable to labour-for-tool exchanges, since most material possessions can be easily purchased with cash from the nearby Matlab bazaar; thus, the effect of cash-for-tool exchange is not significant in our network model. Thus, in a market-integrating region like Matlab, the flow of cash is pivotal in fostering interdependent relationships among households, connecting them across different domains.

However, observed reciprocal exchanges of financial and labour support further hint at the emergence of a ‘patron–client relationship’ in the context of market integration. In such a relationship, the offerings of the patron tend to have greater utility for maintaining livelihoods. Patron–client relationships have long been observed in agricultural (Scott, 1972; Wolf, 1966) and pastoral (Moritz et al., 2011; Pelican, 2012; Sneath, 1993) societies but are often assumed to decline with market integration, as increased access to financial markets reduces clients’ reliance on patrons (Silverman, 1965; Weingrod & Morin, 1971) and/or patrons’ willingness to maintain ties with clients (Scott & Kerkvliet, 1973; Stoler, 1977). Yet in settings like Matlab, where market exchange is widespread but access to formal financial institutions remains limited, cash can become a critical resource that gives rise to a new form of patron–client relationships (Caudell et al., 2015; Rashid, 2012).

### 4.2. Variation in within- and cross-layer reciprocity by socioeconomic status

This leads us to further examine what types of socioeconomic status are closely associated with reciprocal exchanges within and across domains, using motif analysis. Empirical values of within-domain reciprocal ties for landowning, salaried, and political leadership households all fall within the 95% ranges of the simulated values, indicating that these households are not especially more (or less) reciprocal than landless, non-salaried, and non-leader households within any support domain.

These results may be surprising, as reciprocal labour exchanges among farming households are crucial during the busy seasons of planting and harvest. However, in this context, most households rely on multiple sources of subsistence rather than agriculture alone (Erasmus, 1956; Moore, 1975). Similarly, our findings that the households occupying political leadership positions are not more reciprocal within these three support domains differ from some previous studies (Macfarlan et al., 2012; Power & Ready, 2018; Ready & Power, 2018) but are consistent with other studies showing that political leaders are more likely to provide unidirectional support rather than having reciprocal support relationships (Bird & Bliege Bird, 2010; Bliege Bird et al., 2001; Hawkes & Bliege Bird, 2002). In the Matlab context, these results may reflect strong norms of reciprocity widespread in rural Bangladesh (Lynch et al., 2022; Shenk et al., 2021), resulting from high rates of patrilocality and high population density. Thus, even landless and non-leader households could be highly reciprocal with their kin and nearby neighbours with whom they have frequent interactions.

In contrast, salaried households tend to be marginally more reciprocal within empirical financial and labour support layers than they are in simulated networks, although the empirical values are still within the 95% simulated ranges. As an index of greater engagement in the market economy, predictable cash income has often been considered an alternative means for risk reduction that may replace traditional reciprocity-based sharing networks in market-integrated communities (Behrens, 1992; Bohannan, 1959). However, our results provide counterevidence that sharing practices may not simply decline with market integration but rather transform in such a way that cash becomes pivotal in mediating support relationships (Baldassarri, 2020; BurnSilver et al., 2016; Franzen & Eaves, 2007; Gurven et al., 2015; Henrich et al., 2005).

Regarding reciprocity across domains, we find that only salaried households have more reciprocal ties through which one type of support is exchanged for another. When lending money to others, salaried households are more likely to receive material and labour support from those cash-receiving households than what is expected under randomly simulated networks. Closely related to our earlier findings from the multilayered ERGM that the financial and material support layers are reciprocally connected to the labour support layer, this suggests that strong effects of cross-domain reciprocity could be, in fact, driven by salaried households. Salaried households also have more reciprocal ties between material and labour support domains, where they provide material resources and receive labour services. While salaried households have more stable access to cash than their neighbours, salaried workers often work outside Matlab, for example, in nearby cities (Chandpur, Comilla), Dhaka, or even abroad (Mannan, 2016; Rashid, 2012; Siddiqui, 2016; Sznajder et al., 2021). Securing support in childcare and household work from neighbours could be crucial for these households where adult males are absent due to labour migration. As a solution, salaried households can use their wealth to provide financial and material resources to economically insecure households and in return receive help in physical labour, childcare, or other work. This interpretation is consistent with descriptive comparisons showing that salaried households are substantially more likely to include labour migrants than non-salaried households (SI Table 2).

For these reasons, observed cross-domain reciprocity among salaried households could be understood as an adaptive strategy to resolve an asymmetry in the household economy given that material wealth is relatively oversupplied while labour is in short supply. These results also demonstrate how reciprocal exchanges across domains can promote interdependence between households having different comparative advantages. Such interdependence is expected to be mutually beneficial for both cash-providing salaried households and cash-receiving households, as the marginal utility of cash would be higher for the cash-receiving households, while the marginal utility of help in physical labour would be higher for the cash-providing salaried households (Demps & Winterhalder, 2019; Jaeggi et al., 2016; Winterhalder, 1996).

### 4.3. Salaried households as emerging patrons in a market-integrated community

At the same time, however, the patterns of cross-domain reciprocity observed among salaried households indicate that they are potentially emerging as novel patrons in Matlab due to their predictable cash income. Our results show that salaried households are more likely to exchange cash for household items and labour and household items for labour, but not particularly more likely to exchange household items and labour for cash and labour for household items. Of the three types of resources analysed in this study, asymmetries in distribution among Matlab households would be highest for cash, followed by household items and labour. This indicates that salaried households tend to receive less scarce resources (labour, time) in return for providing scarcer resources (cash). While reciprocal relationships across domains would be mutually beneficial for salaried households and their network partners, those providing the scarcer resources would have greater initiative in reciprocal relationships than those providing the less scarce resources. In case of economic crisis, it would be more difficult to find someone who can lend money as only 16 households have predictable cash income in this area. In contrast, salaried households can find help in childcare and physical labour with relative ease, especially in the Matlab context where labour is abundant due to high population density.

Asymmetries in reliance between exchange partners are typical in patron–client relationships as a form of ‘lop-sided friendship’, which explains how mutually beneficial relationships result in social inequality (Eisenstadt & Roniger, 1980). Thus, as people are increasingly relying on cash to sustain livelihoods, salaried households may play a key role in facilitating social exchanges across domains, mainly by providing financial and material support and receiving labour support. As a result, their importance in these support networks may contribute to increased prestige and increasingly lead to patron roles in Matlab.

The potential emergence of salaried households as new patrons can further explain how and why more market-integrated individuals, who may have alternative buffers for risk through stored foods or predictable cash income, are still actively participating in local sharing networks (BurnSilver et al., 2016; Franzen & Eaves, 2007; Gurven et al., 2015). In societies transitioning to join the market economy, storable market foods and cash provide alternative means to buffer risks (Behrens, 1992; Bohannan, 1959), reducing the need to rely on reciprocal sharing networks vulnerable to free riders (Franzen & Eaves, 2007). Thus, food availability may no longer pose a great threat to the lives of people in these transitioning societies, resulting in a decline in food sharing networks. A greater problem, however, often comes from earning cash to purchase market foods, especially in low-income communities (including Matlab) where employment opportunities for well-paid, stable jobs are limited and thus access to cash is highly asymmetric among individuals (Caudell et al., 2015; Fafchamps & Lund, 2003; Little et al., 2014; Townsend, 1994). Under such circumstances, salaried households with predictable cash income have found opportunities to elevate their social standings by generously providing financial support to neighbours in need (Ready & Power, 2018). Findings that social stratification is closely linked with the provisioning of economic support are not themselves novel (Bird & Bliege Bird, 2010; Sahlins, 2013), but our study uniquely illustrates how such mechanisms of social inequality can also operate in the context of market integration.

### 4.4. Decline of landowners and political leaders in local support networks

Landowning households are not more reciprocal across domains in multilayered support networks in Matlab. Landowners served as patrons until the recent past, since agriculture had been the most important source of living for Matlab villagers for centuries (Razzaque & Streatfield, 2002); indeed, large landowners (*zamindars*) are well-known to have been the primary patrons in the region through the mid-20th century when the Partition of India and subsequent land reforms disrupted what was left of the traditional zamindari system (Khan, 2002). Although still constituting a principal mode of livelihood in Matlab for around 40% of households, farming is becoming relatively less profitable (Razzaque et al., 2010; Uddin, 2022) and an increasing proportion of household income comes from those participating in the global market economy system through small businesses, wage labour, education-based salaried jobs, or labour migration (Jackson & Young, 2016; Rashid, 2012; Shenk et al., 2016). In addition, most landowners in modern Matlab do not own large amounts of land, and thus it is perhaps not surprising that they do not generally operate as key links in local status networks the way that large landowners (*zamindars*) may have done in the past (Kabeer, 2001; Phillips et al., 1988). As a result, landowners may be deemed by villagers to be less attractive as patrons or even as network partners in the context of market integration.

Interpretation of the result that households with members in political leadership positions do not have more reciprocal ties across support domains with other households is less straightforward. In the past, these political leaders also tended to serve as patrons in traditional agricultural communities, providing economic support to their clients and in return receiving political and/or labour support from them (Redhead & Power, 2022; Scott, 1972; Wolf, 1966). However, it is possible that enhanced connectivity to national and global systems has provided these political leaders with pre-emptive opportunities to pursue alternative ways of living that they expect will yield greater gains in the long run (Scott & Kerkvliet, 1973; Stoler, 1977). Although patron–client systems assign certain privileges to patrons such as political support and personal services from clients, they also come with obligations as well, which include protecting the well-being of clients and resolving conflicts among clients (Scott, 1972). Thus, rather than continuing to serve as patrons across generations, political leaders may instead have concentrated their resources on developing connections to national and global labour markets and/or to higher authorities. As a result, while still considered to be socially influential by other villagers, political leaders might be much less active in local support networks, allowing salaried workers from non-prestigious families to fill the vacuum in patron positions. Analysis of household connections supports this explanation as households occupying leadership positions have significantly more ties to people outside the village and to social institutions such as government, research institutes, and NGOs (SI Table 4).

### 4.5. Qualitative evidence for emerging social inequalities in Matlab

While our analyses identify cross-layer exchange patterns consistent with emerging patron–client relationships led by salaried households, we acknowledge that these patterns only provide indirect evidence of an emerging patron–client system. Such inferences are limited by the observational nature of our data, which cannot capture the motivations or long-term stability of these exchange relationships. Future research combining longitudinal network data with in-depth qualitative interviews could track how these relationships form, persist, or dissolve over time, while also capturing the perspectives and motivations of those involved in cross-layer exchange relationships.

Yet we have reason to believe that this interpretation is reasonable. Network characteristics of salaried households also indicate that salaried households occupy more central positions in financial (eigenvector) and labour (betweenness) support domains (SI Table 3), suggesting that they may indeed exert greater influence on others in Matlab social networks. Higher hub scores observed among salaried households in the financial support domain further suggest that they financially support neighbours who need the most help (indicated by receiving support from many other households), displaying clear asymmetries in exchanges between cash-providing salaried households and their recipients.

In addition, our interpretation of these results as an emerging patron–client system is supported by qualitative evidence from fieldwork in Matlab, including focus group discussions (FGDs) and informal observations. Across FGDs, participants repeatedly emphasized that the ability to mobilize and distribute money has become increasingly central to earning respect and influence within the context of market integration. As several participants put it,

You better think about the past, you don’t have any money. You don’t have any politics in your mindset. In the previous system the guy who had skill, had fluency in speaking, could do political strikes or meetings. His ability had been used in the politics. But now, they would see your money at first. If don’t have money, you may be involved in the politics, but you won’t get any posts or you won’t have any position in the politics. (Participant in an FGD with 8 young men in Matlab town)

Meanwhile, another participant stressed that ‘Yes, they must have money. They should help the poor. He should help various types of organizations, they must give their valuable time for the society’ (Participant in an FGD with 10 older men in Matlab town). Together, these accounts point to a broader reorganization of local status hierarchies around access to cash, providing a social context in which households with reliable monetary income are positioned to emerge as a new upper tier within village life.

In addition, two of the most widely recognized prestigious households in the study area, headed by former and current village councillors, were frequently named by villagers as respected individuals, yet appeared only rarely in responses to financial and material support questions. When asked about this discrepancy, respondents explained that these households are now largely composed of elderly heads and their spouses, while their adult children, many of whom hold bachelor’s degrees or higher, work in Dhaka or abroad and send remittances home. As a result, these households maintain high social prestige and symbolic authority but are less actively involved in everyday village support exchanges. Villagers emphasized that such households ‘no longer worry much about how people live in the village’, relying instead on remittance income and engaging less in local financial assistance. This distinction illustrates how prestige and leadership status can persist independently of active participation in support networks and highlights a generational and migratory shift in the basis of patronage.

In contrast, younger heads of salaried households, despite their more recent ascent and lower symbolic seniority, were often described as more responsive to local needs and more directly involved in material and financial support. There is an actively operating fundraising committee led by a former village councillor, return labour migrants, and younger salaried workers in the study area, receiving donations to build a mosque in a vacant piece of land that used to be a pond. The inclusion of salaried workers in this committee, despite their being much younger than the other members, suggests their importance in local status networks. Indeed, the number of mosques is increasing quickly in Bangladesh not simply because of economic growth but also because wealthy individuals, especially those who have recently amassed wealth as labour migrants in cities or abroad, very often build new mosques to establish prestige in their home village or bari (Ahamed & Nazneen, 1990; Sabur, 2022).

This pattern was also evident in FGDs. One highly educated return migrant now working as a salaried employee in Matlab explained his local involvement as follows:

Socially and politically, I am so active and work with some political leaders. I am also involved different kind of law related activities in my society. Because of that, I always try to give help to helpless people. We provide rice cards, elderly allowance, widow allowance, lame allowance. We take care of them. (Participant in FGD with 8 young men residing in periurban areas of Matlab)

Thus, additional quantitative and qualitative findings match well with our interpretation of the results that a new patron–client system led by salaried households is emerging in Matlab.

## 5. Conclusion

Drawing on evolutionary models of cooperative partner choice (Barclay & Willer, 2007) and the emergence of social inequality (Mattison et al., 2016; Redhead & Power, 2022; Smith et al., 2023), our study suggests that reciprocal exchanges across support domains can simultaneously foster interdependence among individuals with different comparative advantages and also create conditions under which those with high-value resources become central in exchange networks, potentially emerging as new patrons. Yet these dynamics are not uniform across human societies but instead are likely shaped by the specific socioeconomic contexts in which cooperation occurs (Gurven & Jaeggi, 2015). In the Matlab context, market integration has made predictable cash income more important for everyday survival and exchange than land ownership or political connections, explaining why salaried households are more active in cross-layer reciprocal exchanges while landowning and leadership households are less involved in local support networks. Taken together, these results suggest the emergence of new patron–client dynamics centred on salaried households in a market-integrated community.

Evolutionary models of cooperation and social inequality have largely focused on hunter-gatherers and other subsistence-based communities, but evolutionary researchers are increasingly studying populations undergoing socioeconomic transitions associated with market integration, as truly isolated communities are now exceedingly rare (Sear et al., 2024). Our study demonstrates that evolutionary frameworks remain relevant for understanding cooperation and social inequality in market-integrated contexts. By identifying domains of exchange that are critical under new economic conditions (i.e., financial support) and the socioeconomic status(es) most likely to access those resources (i.e., salaried jobs), we show how partner choice and asymmetric access to resources continue to shape reciprocity and interdependence in ways consistent with evolutionary theory. Rather than treating market integration as a departure from conditions in which evolutionary models apply, our analysis highlights how these models can be extended and refined to capture the dynamics of cooperation and inequality in rapidly transforming communities.

## Supporting information

10.1017/ehs.2026.10061.sm001 Hwang et al. supplementary material Hwang et al. supplementary material

## Supplementary material

The supplementary material for this article can be found at https://doi.org/10.1017/ehs.2026.10061.

## Research transparency and reproducibility interest

All the de-identified data and the R codes necessary for replicating the results of this paper are publicly available on Joon Hwang’s GitHub repository (https://github.com/JoonHwang-psu/MatlabMultinetwork).

## 1 Long description

The diagram consists of three sections labeled a, b and c. Section a is titled ′Within-layer reciprocity′ and shows three pairs of houses connected by arrows. The first pair is connected by solid arrows indicating financial support, the second by double-lined arrows indicating material support and the third by dashed arrows indicating labor support. Section b is titled ′Cross-layer reciprocity′ and shows three pairs of houses connected similarly, with solid, double-lined and dashed arrows representing financial, material and labor support, respectively. Section c is titled ′Cross-layer reciprocity depending on socioeconomic statuses′ and includes houses in different colors: green for landowning households, blue for salaried households and yellow for leadership households. Each pair of houses is connected by arrows representing financial, material and labor support. The legend explains the types of support and household statuses: solid arrows for financial support, double-lined arrows for material support, dashed arrows for labor support, green for landowning households, blue for salaried households and yellow for leadership households.

Navigate back to 1.

## Figure 2 Long description

The image consists of three network layers depicting support among households. The first layer is the Financial Support Network Layer, the second is the Material Support Network Layer and the third is the Labor Support Network Layer. Each layer shows nodes representing households connected by directed edges indicating support relationships. The nodes are color-coded: landowning households, salaried households, leadership households, landowning plus salaried, landowning plus leadership, salaried plus leadership and households without all three. The connections illustrate different types of support provided and received among the households.

Navigate back to Figure 2.

## Figure 3 Long description

The image contains nine histograms showing reciprocal ties within layers. Each row represents a different socioeconomic status: land ownership, salaried income and political leadership. Each column represents a different support layer: financial, material and labor. The x-axis represents the number of reciprocal ties and the y-axis represents frequency. The light blue bars indicate the 95 percent simulated range, while the dark vertical line represents the empirical value. 1. Financial support layer, land ownership: The empirical value is near the center of the distribution at 31, with a peak around 31. 2. Material support layer, land ownership: The empirical value is at 67, near the center of the distribution, with a peak around 67. 3. Labor support layer, land ownership: The empirical value is at 11, near the center, with a peak around 11. 4. Financial support layer, salaried income: The empirical value is at 27, near the center, with a peak around 27. 5. Material support layer, salaried income: The empirical value is at 42, near the center, with a peak around 42. 6. Labor support layer, salaried income: The empirical value is at 11, near the center, with a peak around 11. 7. Financial support layer, political leadership: The empirical value is at 9, near the center, with a peak around 9. 8. Material support layer, political leadership: The empirical value is at 21, near the center, with a peak around 21. 9. Labor support layer, political leadership: The empirical value is at 6, near the center, with a peak around 6. Overall, the empirical values generally fall near the center of the simulated distributions across all layers and socioeconomic statuses, indicating consistency between empirical and simulated data.

Navigate back to Figure 3.

## Figure 4 Long description

The title reads Reciprocal ties across layers. The composite contains 18 histograms arranged in 6 rows and 3 columns. Rows represent three socioeconomic statuses: land ownership, salaried income and political leadership. Columns represent six cross-layer support pairings: providing financial support and receiving material support, providing financial support and receiving labor support, providing material support and receiving labor support, providing material support and receiving financial support, providing labor support and receiving financial support and providing labor support and receiving material support. Each histogram shows bars representing the 95 percent simulated range and a vertical line representing the empirical value. The x-axis in each histogram represents the count of reciprocal ties, with values grouped in intervals of approximately 1 to 3 units. The y-axis represents the frequency count of simulations, ranging from 0 to approximately 100 to 140 depending on the panel. The legend identifies the bars as 95 percent simulated range and the vertical line as empirical value. Land ownership row: For providing financial support and receiving material support, the x-axis extends from 16 to 55, the distribution peaks near 28 to 32 and the empirical line falls near 32. For providing financial support and receiving labor support, the x-axis extends from 5 to 36, the distribution peaks near 16 to 20 and the empirical line falls near 18. For providing material support and receiving labor support, the x-axis extends from 9 to 44, the distribution peaks near 22 to 26 and the empirical line falls near 24. For providing material support and receiving financial support, the x-axis extends from 16 to 51, the distribution peaks near 30 to 36 and the empirical line falls near 36. For providing labor support and receiving financial support, the x-axis extends from 9 to 31, the distribution peaks near 18 to 22 and the empirical line falls near 20. For providing labor support and receiving material support, the x-axis extends from 12 to 42, the distribution peaks near 24 to 28 and the empirical line falls near 27. Salaried income row: For providing financial support and receiving material support, the x-axis extends from 7 to 32, the distribution peaks near 16 to 20 and the empirical line falls near 30, toward the far right of the simulated range. For providing financial support and receiving labor support, the x-axis extends from 2 to 23, the distribution peaks near 10 to 14 and the empirical line falls near 20, toward the right edge of the simulated range. For providing material support and receiving labor support, the x-axis extends from 4 to 29, the distribution peaks near 14 to 18 and the empirical line falls near 25, toward the right of the peak. For providing material support and receiving financial support, the x-axis extends from 8 to 34, the distribution peaks near 18 to 22 and the empirical line falls near 27 to 28, near the right edge. For providing labor support and receiving financial support, the x-axis extends from 2 to 25, the distribution peaks near 10 to 14 and the empirical line falls near 18 to 19. For providing labor support and receiving material support, the x-axis extends from 3 to 30, the distribution peaks near 13 to 17 and the empirical line falls near 19. Political leadership row: For providing financial support and receiving material support, the x-axis extends from 4 to 31, the distribution peaks near 10 to 14 and the empirical line falls near 12. For providing financial support and receiving labor support, the x-axis extends from 1 to 23, the distribution peaks near 8 to 12 and the empirical line falls near 9. For providing material support and receiving labor support, the x-axis extends from 1 to 27, the distribution peaks near 10 to 14 and the empirical line falls near 12. For providing material support and receiving financial support, the x-axis extends from 4 to 27, the distribution peaks near 8 to 12 and the empirical line falls near 9. For providing labor support and receiving financial support, the x-axis extends from 1 to 18, the distribution peaks near 8 to 10 and the empirical line falls near 9. For providing labor support and receiving material support, the x-axis extends from 2 to 25, the distribution peaks near 10 to 13 and the empirical line falls near 15 to 16, slightly to the right of the peak. The distributions in the land ownership and political leadership rows are generally bell-shaped and the empirical lines fall near or within the central peak region. In the salaried income row, the empirical lines fall consistently toward the right portion of the simulated distributions, notably in the providing financial support and receiving material support and providing financial support and receiving labor support panels.

Navigate back to Figure 4.
