# Supplementary material for: Reciprocal exchanges across multilayered networks show an emerging patron–client system led by salaried households
Source: Evol Hum Sci. 2026 Jul 3;8:e29. doi: 10.1017/ehs.2026.10061 (PMC13420160; doi:10.1017/ehs.2026.10061)
Supplement: Hwang et al. supplementary material [file S2513843X26100619sup001.docx]

**Supplementary Information**

**Household material wealth survey**

The household wealth is measured through quantification of the number of household property holdings and its translation into monetary values, in this study, in US dollars. The values of houses and other assets are estimated with depreciation, and then summed to produce total estimated household wealth. The list of house characteristics and other assets included for calculating wealth is as follows:

*House characteristics:*

1) house ownership / inheritance 2) number of houses owned

3) age of main house (for calculating depreciation) 4) number of rooms

5) material of construction for roof / wall / floor 6) type of stove fuel

7) ownership and type of toilet

*Household assets:*

1) torch 2) fan 3) electric light 4) solar array

5) generator 6) TV 7) refrigerator 8) blanket

9) mattress 10) cot 11) chair 12) sofa

13) sofa set 14) storage rack 15) cabinet 16) table

17) dressing table 18) pot 19) chicken / duck 20) goat

21) cow 22) rickshaw 23) autorickshaw 24) bicycle

25) motorcycle 26) CNG / tomtom 27) boat

28) shop 29) tractor 30) tubewell 31) pond

32) mobile phone / smart phone / laptop

**Social support network survey**

Social network data result from a survey conducted with the villagers. Using the ‘name generator’ approach^1^, respondents were asked to free-list individuals in 12 questions and to give sociodemographic information on those individuals so that we could match them to the same individuals in other respondents’ networks. In Question 1 and 2, they named individuals whom they would borrow money from (Q1), and whom they would lend money to in case of emergency (financial support network). In Question 3 and 4, they named individuals whom they would borrow food items or small household items from (Q3) and whom they would lend these items to (Q4) (material support network). In Question 5 and 6, they named individuals whom they would ask for help when female household members need help caring for their children or grandchildren (Q5) and when male household members need help with work in the house or community (Q6) (labor support network). In Question 7 and 8, they named individuals whom female members (Q7) and male members (Q8) usually spend time talking with or visiting. In Question 9, they named government workers, ICDDRB employees, or other NGO employees they can ask for help, and in question 10, they named the most important ties outside of their village. In Question 11 and 12, they named individuals whom female members (Q11) and male members (Q12) normally work with. Since this study uses household as basic unit of analysis, we merged the responses if the respondents named two different individuals in the same household without any weights, so that the resulting support network is unweighted, simply indicating presence and absence of support relationships between the households. Theoretically, 12 different questions can generate 12 different layers of a multilayered network. However, for actual analysis, only first six questions are used, because social support relationships, the main focus of this study, were evident only in these questions. Furthermore, two closely related questions are merged so that they generate a combined layer of the same support type. As a result, the financial support layer was generated by merging money borrowing and lending questions (Q1&2), the material support layer by merging the food/household items borrowing and lending questions (Q3&4), and the labor support layer by merging the women’s childcare and men’s help networks (Q5&6). In the resulting combined layers, the direction of all ties reflects the direction of resource flow, regardless of whether the donor or recipient nominated the relationship.

**Descriptive statistics of household demographic and socioeconomic attributes**

SI Table 1 illustrates descriptive statistics of the household demographic and socioeconomic attributes. SI Table 2 compares these attributes between households with and without land ownership, salaried income, and political leadership positions. Salaried households had higher educational attainment and were more likely to include labor migrants than non-salaried households. Landowning households had greater household wealth than non-landowning households, while households with political leadership positions had higher educational attainment and household wealth than non-leadership households.

Differences in network characteristics depending on household socioeconomic status (land ownership, salaried income, and political leadership) are shown in SI Table 3. Due to highly skewed distribution of network characteristics among the households, Wilcoxon rank-sum test was used to test how network characteristics vary depending on a status type. The number of support-receiving (in-degree) and support-providing ties (out-degree) are compared between the households with and without certain status types, to examine differences in degree centrality. Unlike degree centrality, structural centrality measures (eigenvector and betweenness centrality) account for network structures that extend beyond direct relationships between two nodes. Eigenvector centrality measures the importance that a target node has in a network, by considering the connectivity of the nodes connected to the target node^2^. Betweenness centrality, on the other hand, measures how often an individual node provides the short path connecting other nodes^3,4^. Higher centrality in network structures in general indicates that the node has greater importance or influence over others in social networks, by having connections to other influential nodes (eigenvector) or by connecting two or more nodes otherwise unconnected to each other (betweenness). Finally, we adopted Hyperlink-Induced Topic Search (HITS) algorithm^5^ to evaluate hub and authority scores of the respondent households in support layers. Although originally developed to explore the importance of web pages as information source in the context of World Wide Web, hub and authority are useful for investigating directed social networks where the hub has outgoing ties to nodes linked by many other nodes and the authority has incoming ties from nodes that link to many other nodes. In the context of support network, higher hub scores indicate that the target node supports other nodes that receive support from many others, while higher authority scores indicate that the target node is supported by other nodes that provide support to many others. The number of ties respondent households have with people outside the study area (“External ties”) and to social institutions (“Institutional ties”) is shown in SI Table 4. While these connections may have important implications regarding geographic expansiveness and social influence of network actors, they could not be included in the analysis of local support networks since they do not represent social relationships between local villagers. Thus, we counted the number of names given by the respondents for Q9 (institutional ties) and 10 (external ties) of the network survey and summarized how such connections vary depending on socioeconomic status of the households. Wilcoxon rank-sum test was used to test if the differences between households with and without certain status types are significant.

***SI Table 1. Descriptive statistics of the household-level variables***

| Statistic | N | Mean | St. Dev. | Min | Max |
| --- | --- | --- | --- | --- | --- |
| Household size | 79 | 4.19 | 1.70 | 1 | 8 |
| Household head age | 79 | 51.20 | 15.00 | 24 | 85 |
| Household wealth (USD) | 79 | 2,657.84 | 1,527.86 | 407 | 8,023 |
| Land ownership (n=29) | 79 | 0.37 | 0.49 | 0 | 1 |
| Salaried income (n=16) | 79 | 0.20 | 0.40 | 0 | 1 |
| Political leadership (n=9) | 79 | 0.11 | 0.32 | 0 | 1 |

***SI Table 2. Demographic and socioeconomic characteristics of the households depending on socioeconomic status***

| Mean (SD)  or n (%) | | Household size | Mean age | Household head age | Mean education | Highest education | Household  wealth (USD) | Labor migrant (%) |
| --- | --- | --- | --- | --- | --- | --- | --- | --- |
| **Land ownership** | Yes  (n=29) | 4.41  (1.86) | 34.66  (11.57) | 55.34  (13.40) | 3.18  (1.05) | 4.14  (1.43) | 3214  (1752) | 7  (24.1%) |
|  | No  (n=50) | 4.06  (1.61) | 31.17  (15.48) | 48.80  (15.47) | 2.93  (1.29) | 3.86  (1.46) | 2336  (1294) | 15  (30.0%) |
|  | *p ^†^* | 0.398 | 0.053 **∙** | 0.073 **∙** | 0.369 | 0.407 | 0.036 * | 0.614 |
| **Salaried income** | Yes  (n=16) | 4.31  (1.40) | 29.43  (12.38) | 45.31  (13.81) | 3.65  (1.04) | 4.56  (0.89) | 2500  (1228) | 11  (68.8%) |
|  | No  (n=63) | 4.16  (1.78) | 33.22  (14.61) | 52.70  (15.02) | 2.86  (1.20) | 3.81  (1.52) | 2698  (1601) | 11  (17.5%) |
|  | *p* | 0.659 | 0.300 | 0.081 **∙** | 0.041 * | 0.056 **∙** | 0.927 | <0.001 ** |
| **Political leadership** | Yes  (n=9) | 4.78  (2.33) | 36.78  (9.99) | 58.44  (14.18) | 3.71  (0.97) | 5.00  (1.00) | 4239  (2376) | 2  (22.2%) |
|  | No  (n=70) | 4.11  (1.61) | 31.89  (14.61) | 50.27  (14.94) | 2.93  (1.21) | 3.83  (1.44) | 2454  (1271) | 20  (28.6%) |
|  | *p* | 0.446 | 0.121 | 0.293 | 0.022 * | 0.019 * | 0.019 * | 1.000 |

*Note: † Wilcoxon rank-sum tests (all variables) and Fisher's exact tests (labor migrant households)*

***∙*** *p<0.1; * p<0.05; ** p<0.01*

| ***SI Table 3. Network characteristics of the households depending on socioeconomic status*** | | | | | | | | | | | | | |
| --- | --- | --- | --- | --- | --- | --- | --- | --- | --- | --- | --- | --- | --- |
| *Network layer* |  | *Degree centrality* | | | | *Structural centrality* | | | | *HITS algorithm* | | | |
| **Financial support** |  | In | *p* | Out | *p* | Eigenvector | *p* | Betweenness | *p* | Hub | p | Authority | *p* |
| Land ownership | Yes | 2.10 | 0.50 | 2.28 | 0.65 | 0.16 | 0.23 | 0.02 | 0.55 | 0.06 | 0.62 | 0.06 | 0.47 |
|  | No | 2.40 |  | 2.30 |  | 0.26 |  | 0.02 |  | 0.08 |  | 0.08 |  |
| Salaried income | Yes | 2.50 | 0.49 | 3.13 | <0.01 | 0.34 | 0.07 | 0.02 | 0.70 | 0.12 | <0.01 | 0.09 | 0.26 |
|  | No | 2.24 |  | 2.08 | ** | 0.19 | ∙ | 0.02 |  | 0.06 | ** | 0.07 |  |
| Political leadership | Yes | 1.56 | 0.17 | 3.00 | 0.11 | 0.15 | 0.23 | 0.02 | 0.98 | 0.11 | 0.21 | 0.05 | 0.73 |
|  | No | 2.39 |  | 2.20 |  | 0.23 |  | 0.02 |  | 0.07 |  | 0.08 |  |
|  |  |  |  |  |  |  |  |  |  |  |  |  |  |
| **Material support** |  | In | *p* | Out | *p* | Eigenvector | *p* | Betweenness | *p* | Hub | *p* | Authority | *p* |
| Land ownership | Yes | 2.83 | 0.59 | 3.00 | 0.74 | 0.20 | 0.97 | 0.04 | 0.93 | 0.07 | 0.22 | 0.06 | 0.45 |
|  | No | 3.16 |  | 3.06 |  | 0.22 |  | 0.04 |  | 0.06 |  | 0.06 |  |
| Salaried income | Yes | 3.19 | 0.47 | 3.94 | 0.02 | 0.21 | 0.79 | 0.05 | 0.27 | 0.07 | 0.99 | 0.06 | 0.65 |
|  | No | 3.00 |  | 2.81 | * | 0.21 |  | 0.04 |  | 0.06 |  | 0.06 |  |
| Political leadership | Yes | 2.67 | 0.43 | 3.67 | 0.47 | 0.18 | 0.27 | 0.03 | 0.64 | 0.10 | 0.53 | 0.09 | 0.37 |
|  | No | 3.09 |  | 2.96 |  | 0.22 |  | 0.04 |  | 0.05 |  | 0.06 |  |
|  |  |  |  |  |  |  |  |  |  |  |  |  |  |
| **Labor support** |  | In | *p* | Out | *p* | Eigenvector | *p* | Betweenness | *p* | Hub | *p* | Authority | *p* |
| Land ownership | Yes | 1.62 | 0.56 | 1.69 | 0.86 | 0.23 | 0.70 | 0.01 | 1.00 | 0.03 | 0.66 | 0.05 | 0.59 |
|  | No | 1.80 |  | 1.76 |  | 0.26 |  | 0.01 |  | 0.07 |  | 0.06 |  |
| Salaried income | Yes | 2.13 | 0.20 | 2.19 | 0.22 | 0.32 | 0.14 | 0.01 | 0.07 | 0.10 | 0.14 | 0.09 | 0.82 |
|  | No | 1.63 |  | 1.62 |  | 0.23 |  | 0.01 | ∙ | 0.05 |  | 0.05 |  |
| Political leadership | Yes | 1.56 | 0.63 | 3.22 | 0.04 | 0.21 | 0.53 | 0.02 | 0.43 | 0.17 | <0.01 | 0.03 | 0.32 |
|  | No | 1.76 |  | 1.54 |  | 0.26 |  | 0.01 |  | 0.04 | ** | 0.06 |  |
| *Note:*  ***∙*** *p<0.1; * p<0.05; ** p<0.01* | | | | | | | | | | | | | |

***SI Table 4. The number of household ties to people outside the village and to social institutions***

|  |  | External ties | *p* | Institutional ties | *p* |  |
| --- | --- | --- | --- | --- | --- | --- |
| Land ownership | 1 | 7.10 | 0.59 | 1.34 | 0.57 |  |
|  | 0 | 7.62 |  | 1.30 |  |  |
| Salaried income | 1 | 9.31 | 0.09 | 1.21 | 0.40 |  |
|  | 0 | 6.95 | ∙ | 1.75 |  |  |
| Political leadership | 1 | 10.33 | 0.02 | 2.78 | 0.03 |  |
|  | 0 | 7.06 | * | 1.13 | * |  |
| *Note:*  ***∙*** *p<0.1; * p<0.05; ** p<0.01* | | | | | | |

**Multilayered Exponential Random Graph Model**

We applied Multilayered Exponential Random Graph Model (ERGM) to fit the fundamental model to our multilayered support network, using the *statnet*^6^ and *ergm.multi*^7^ package in R 4.3.2^8^. In ERGM, the probabilities that two nodes have a tie depending on their own characteristics (nodal attributes) and their relationships (relational attributes) with other nodes are estimated using maximum pseudolikelihood estimator and Markov Chain Monte Carlo (MCMC) algorithms^9^. One distinct feature of the multilayered ERGM is that it can calculate such probabilities when the nodes have ties with others across multiple network layers by modeling the marginal dependence among multiple layers based on Conway-Maxwell-Binomial distribution^7^.

Results from the multilayered ERGM are shown in SI Table 5. The first section “All layers combined” shows that we calculate how kinship and geographic distances between households affect tie probabilities in the empirical network when all support layers are combined. In other words, the odds ratios in this section show how likely that two households have a tie of any support type when they are related through kinship, or when the distance between them increases by 100m. The results that the tie probability increases with kinship and decreases with physical distance correspond well with our initial intuition that such geosocial constraints would have effects on social relationships regardless of the support types.

In the following three sections, the tie probability is calculated in each domain of support. Thus, the variables included in these sections are conceptually identical, but they produce different outcomes as they predict the presence of a tie in different layers. It should be noted that unlike other geographic and social constraints such as kinship and distance, wealth difference between the households is employed not in a combined network space but in each support layer, because material wealth is expected to affect tie formation only in support domains that are directly related to household economies (i.e., financial and material support), but not in others (i.e. labor support).

***SI Table 5. Results from the network model fitted through multilayered ERGM.***

| Odds Ratio (95% CI) | Tie probability  in multilayered networks | |
| --- | --- | --- |
| ***All layers combined*** |  |  |
| Distance (100m) | 0.823 | (0.766, 0.884) |
| Kinship | 1.213 | (1.078, 1.366) |
| ***Financial support layer*** |  |  |
| Edges | 0.011 | (0.008, 0.015) |
| Isolates ^a^ | 0.581^†^ | (0.135, 2.506) |
| Transitive triads ^b^ | 1.089 | (0.812, 1.461) |
| Reciprocity | 12.394 | (5.482, 28.018) |
| Wealth difference ^c^ | 1.171 | (1.062, 1.290) |
| ***Material support layer*** |  |  |
| Edges | 0.008 | (0.006, 0.011) |
| Isolates | 0.691 | (0.161, 2.977) |
| Transitive triads | 1.535 | (1.258, 1.873) |
| Reciprocity | 132.298 | (65.682, 266.476) |
| Wealth difference | 1.129 | (1.006, 1.267) |
| ***Labor support layer*** |  |  |
| Edges | 0.010 | (0.007, 0.014) |
| Isolates | 0.320 | (0.075, 1.368) |
| Transitive triads | 2.653 | (1.755, 4.011) |
| Reciprocity | 0.225 | (0.076, 0.660) |
| Wealth difference | 1.025 | (0.942, 1.115) |
| ***Cross-layer dependencies*** |  |  |
| *Same direction ties in two layers* |  |  |
| Financial & Material | 24.406 | (13.007, 45.793) |
| Financial & Labor | 2.336 | (1.166, 4.678) |
| Material & Labor | 3.025 | (1.363, 6.711) |
| *Cross-layer reciprocity* |  |  |
| Financial - Material | 0.562 | (0.265, 1.194) |
| Financial - Labor | 3.768 | (1.990, 7.135) |
| Material - Labor | 6.712 | (3.142, 14.340) |

*Note:* *^a^ the number of nodes not connected to any other nodes*

*^b^ the number of nodes having one transitive shared partner*

^c^ *(donor’s wealth in USD – recipient’s wealth in USD) /1000*

*^†^ Odd Ratios not significantly different from 1 are colored light grey*

The term “Edges” counts the number of ties in each support layer and controls the density of a single layer network so that the simulated networks do not deviate too far from the empirical network in terms of density. The terms “Isolates” and “Transitive triads” are additional structural constraints adopted in the fundamental model to improve the goodness-of-fit of the fundamental model. To be specific, the term “Isolates” counts the number of nodes that are not connected to any other nodes in each support layer (SI Figure 1, right). Such control for the isolates helps improve goodness-of-fit of the simulated networks in terms of geodesic distance even without any significant effects on tie formation in all three layers, as it prevents simulated networks from being over-connected, unduly exceeding the level of connectivity observed in empirical networks. For the transitive triads, we only count the case where two nodes *u* and *v* that are connected through the edge (u, v) also have the edges {(u, w), (w, v)} but only once with the third node *w* (SI Figure 1, left), which is equivalent to one edgewise shared partner (“esp(1)” in R *ergm* package). Significant effects of transitive triads in material and labors support layers indicate that if household *u* supports *w* and *w* supports *v* (SI Figure 1, left), household *u* is 1.5 and 2.6 times as likely to provide material and labor support to household *v*. We choose to use this instead of geometrically weighted edgewise shared partnerships (GWESP) that are often employed to account for transitivity in the ERGM-based network analysis, since our empirical networks are sparsely connected and thus the nodes rarely have two or more edgewise shared partners. By using isolates and transitive triads as such structural controls of the fundamental model, we could obtain good fit for the simulated networks, especially in terms of geodesic distance (SI Figure 5) and edgewise shared partners (SI Figure 6). In the iterative processes of building a fundamental model, we try to limit structural controls as minimal as possible to avoid overfitting. Also, while introducing excessive structural controls for one network dimension may improve the goodness-of-fit for that dimension, it often produced greater problems in other dimensions.

***SI Figure 1. Transitive triad (left) and isolated node (right) in social networks***

The term “Reciprocity” counts the number of mutual ties between the nodes in each support layer. It is of high importance for our study to control mutual support relationships, since it serves multiple functions in the fundamental model. First, it adjusts double sampling structure of the network interview which asked respondents to give names of individuals who support them (support reception) and whom they support (support provision). Such double sampling of the network responses is frequently used in the name generator network survey^10–12^ to deal with bias and inaccuracy in subjective reporting of network partners, but it also entails the risk of inflating reciprocal relationships if the respondents are biased to list the same individual in both support reception and support provision questions^13^. In fact, we observe large increases in the odds of within-layer reciprocity in financial (OR=12.394) and material support layers (OR=132.298). Inclusion of the reciprocity term in the fundamental model can constrain the simulated networks to reflect double-sampled nature of our network survey. By controlling reciprocity in the fundamental model, we can make sure that such potential inflation in the odds of within-layer reciprocity in the fundamental model does not weaken the validity of our main results from motif analysis. Second, by controlling overall tendency of being reciprocal among general households regardless of their socioeconomic status, this term provides a more accurate basis for comparing status-labeled reciprocity in motif analysis. For example, Matlab households may truly have strong reciprocal relationships with other households in general, irrespective of the double sampling. If this is the case, the fundamental model without such controls for reciprocity will simulate networks that are overall much less reciprocal than the empirical network and the simulated range of the status-labeled reciprocity motifs will be far below the empirical motif values. This is not because socioeconomic statuses have strong influence on having reciprocal relationships, but because all the nodes in the simulated networks have smaller number of reciprocal ties than those in the empirical network. Thus, including the reciprocity term makes our simulated networks more closely resemble the empirical network in terms of reciprocity, thereby improving the validity of motif analysis. Finally, within-layer reciprocity term helps the fundamental model more accurately estimate the effects of cross-layer reciprocity. The specific logic behind this will be discussed in the following paragraph.

In the section named “Cross-layer dependencies,” we calculate how the network ties in one layer affect the probabilities of a tie in another layer. Two types of tie configuration are possible when two nodes interact with each other in two layers of a directed network. One is the same direction ties where one node has incoming or outgoing ties with another node in two different layers (SI Figure 2a), indicating that one household receives (provides) two different types of support from (to) another household in our study context. The other is reciprocal ties across layers where one node has an incoming (outgoing) tie with another node in one layer, while having an outgoing (incoming) tie in a different layer (SI Figure 2b), indicating that two households trade one type of support for another. While the latter is directly related to our research interest but the former is not, the term on same direction ties across layers is required in order to accurately estimate the effects of multilayered reciprocity, since having reciprocal relationships within two different support layers can be counted as both two same direction ties and reciprocal ties across layers (SI Figure 2c), confounding the effects of multilayered reciprocity. Thus, by controlling both reciprocity within layers and the same direction ties across layers^7^, the estimated effects of multilayered reciprocity can truly represent whether Matlab households are more likely to exchange one type of support for another. The multilayered ERGM results indicate that having a directed tie in one support layer significantly increases the probabilities of having the same direction ties in other two layers, but the effects of cross-layer reciprocity are also significant between financial and labor support domains and between material and labor support domains even after controlling for the effects of within-layer reciprocity and the same direction ties across layers (SI Table 5).

***SI Figure 2. Different forms of tie configuration in multilayered network space***

**Goodness-of-fit of the fundamental model**

Finally, we conducted goodness-of-fit diagnostics to test the correspondence in network statistics between the empirical and synthetic networks simulated from the fundamental model. We used the method proposed by Hunter et al.^14^ to test the goodness-of-fit of the ERGMs using key network properties including in- and out-degrees, geodesic distances and edgewise shared partners. The goodness-of-fit of the fundamental model is of particular importance to our study, since the results we obtained from motif analysis would be critically weakened if the synthetic networks fail to reproduce these general network characteristics of the empirical network. The goodness-of-fit was tested for each support layer separately, but not for the entire multilayered network, because even if simulated multilayered networks may show good fit overall, network features in each layer can critically differ between the simulated and empirical networks. Thus, layer-specific testing of the goodness-of-fit helps us ensure that the simulated networks well represent support relationships in different domains, improving the validity of our motif analysis.

In- and out-degree count how many incoming and outgoing ties each node has in social networks. SI Figure 3 and 4 show that the network statistics of the empirical networks (black line) fall within the 95% range of those from the simulated networks (grey line), while some values moderately deviate from the mean simulated values (blue diamond). This indicates that the fundamental model successfully simulates synthetic networks that resemble the empirical network in terms of in- and out-degree distribution. SI Table 6 and 7 further illustrate specific in- and out-degree distribution of the empirical network (“obs.”) and synthetic networks (“min”, “mean”, “max”), and Monte Carlo (MC) p-values^15^ testing whether the empirical in- and out-degree values significantly differ from the simulated values from the Monte Carlo randomization. Since our synthetic networks are already simulated based on MCMC algorithms, we could obtain MC p-values by calculating the probability of observing the simulated values at least as extreme as the empirical values in two-tailed test format, since we cannot pre-emptively decide whether the simulated values will be larger or smaller than the empirical values. All the MC p-values for in- and out-degree comparison are above 0.05, so we could not reject the null hypothesis that in- and out-degree distribution is identical between the empirical and simulated networks at a significance level of 0.05.

Geodesic distance measures the length of the shortest path between two nodes. If two nodes are connected only through the third node, their geodesic distance is 2. The goodness-of-fit diagnostics show that the empirical distribution of geodesic distances is observed within the 95% range of the synthetic distribution (SI Figure 5), and the MC p-values also indicate that all the geodesic distance frequencies observed in the empirical network are not significantly (*p*>0.05) different from those in the synthetic networks (SI Table 8). Another point to be noted here is that the geodesic distance can be infinite, if one node cannot reach another through the existing ties. Thus, if some nodes are isolated, completely disconnected from others, they will have infinite geodesic distances to all other nodes. Since the presence of isolated nodes can substantially increase the frequencies of infinite geodesic distance, we constrained the number of isolated nodes in the fundamental model to prevent synthetic networks from being over-connected and thus deviating far from the empirical network in geodesic distance distribution (SI Table 6, “Inf”).

Edgewise shared partners mean that two nodes at the endpoints of a tie have ties with other nodes. Such tie configuration is analogous to transitive relationships where a person becomes a friend of his or her friend. Thus, if these two individuals share two friends in common, they have two edgewise shared partners. Due to the sparse connectivity of the empirical network, however, most of the nodes in the empirical network have no edgewise shared partner at all or have only one, while 2 or 3 edgewise shared partners are very rarely observed in all three layers (SI Table 9). This empirical network characteristic allows us to control transitivity through the ties having only one edgewise shared partner (“esp(1)” in R *ergm* package) in the fundamental model, instead of GWESP that requires somewhat artificial decay parameter and often results in degeneracy. Such controls for one edgewise shared partner help improve the goodness-of-fit of the simulated networks in cases of zero and one edgewise partner, but the simulated networks start to deviate from the empirical network for two and three edgewise shared partners in financial and material support layers (SI Figure 6). MC p-values also indicate that the counts of two edgewise shared partners in financial support layer, and the counts of two and three edgewise shared partners in material support layers significantly differ between the empirical and simulated networks (*p*<0.05). Such deviations are undesirable, but we decided not to include additional control terms for two or more edgewise shared partners (“esp(2)” or “esp(3)”) in the fundamental model, since we did not want to arbitrarily constrain simulated networks just to fit the empirical network without any theoretical justification, especially given that two or more edgewise shared partners only comprise relatively small proportion of ties in financial and material support layers. It should be noted that we choose to control for one edgewise shared partners, not just to make simulated networks look closer to the empirical network. This decision was rather based on established network theories on transitivity^16^ and our observation that transitive relationships with more than two edgewise shared partners are rare in the empirical network.

***SI Figure 3. In-degree distribution of the empirical network (black line) vs. simulated networks (box plot)***

***SI Table 6. In-degree comparison between the empirical network (Obs.) and simulated networks (Min., Mean, Max.) and Monte Carlo (MC) p-values of the comparison***

*1) Financial support layer 2) Material support layer 3) Labor support layer*

|  | Obs. | Min. | Mean | Max. | MC p-value |  |  | Obs. | Min. | Mean | Max. | MC p-value |  |  | Obs. | Min. | Mean | Max. | MC p-value |
| --- | --- | --- | --- | --- | --- | --- | --- | --- | --- | --- | --- | --- | --- | --- | --- | --- | --- | --- | --- |
| 0 | 13 | 3 | 10.402 | 23 | 0.528 |  | 0 | 3 | 0 | 5.878 | 19 | 0.396 |  | 0 | 16 | 6 | 16.382 | 27 | 1.000 |
| 1 | 15 | 10 | 20.806 | 35 | 0.216 |  | 1 | 14 | 5 | 14.372 | 30 | 1.000 |  | 1 | 23 | 14 | 25.560 | 40 | 0.660 |
| 2 | 19 | 10 | 19.616 | 30 | 0.980 |  | 2 | 15 | 7 | 16.976 | 30 | 0.692 |  | 2 | 15 | 8 | 18.462 | 29 | 0.428 |
| 3 | 15 | 4 | 13.340 | 24 | 0.700 |  | 3 | 22 | 2 | 15.072 | 26 | 0.088 |  | 3 | 16 | 3 | 10.120 | 22 | 0.112 |
| 4 | 7 | 0 | 7.530 | 19 | 1.000 |  | 4 | 10 | 2 | 11.348 | 24 | 0.824 |  | 4 | 7 | 0 | 4.672 | 11 | 0.368 |
| 5 | 6 | 0 | 3.694 | 12 | 0.392 |  | 5 | 5 | 0 | 7.044 | 18 | 0.656 |  | 5 | 1 | 0 | 1.796 | 8 | 0.944 |
| 6 | 2 | 0 | 1.634 | 6 | 0.952 |  | 6 | 4 | 0 | 3.872 | 11 | 1.000 |  | 6 | 0 | 0 | 0.708 | 5 | 1.000 |
| 7 | 0 | 0 | 0.670 | 5 | 1.000 |  | 7 | 3 | 0 | 1.974 | 8 | 0.616 |  | 7 | 0 | 0 | 0.224 | 2 | 1.000 |
| 8 | 1 | 0 | 0.232 | 4 | 0.392 |  | 8 | 2 | 0 | 0.850 | 5 | 0.408 |  | 8 | 0 | 0 | 0.062 | 1 | 1.000 |
| 9 | 0 | 0 | 0.056 | 2 | 1.000 |  | 9 | 0 | 0 | 0.388 | 3 | 1.000 |  | 9 | 0 | 0 | 0.006 | 1 | 1.000 |
| 10 | 0 | 0 | 0.016 | 1 | 1.000 |  | 10 | 0 | 0 | 0.160 | 2 | 1.000 |  | 10 | 0 | 0 | 0.008 | 1 | 1.000 |
| 11 | 0 | 0 | 0.004 | 1 | 1.000 |  | 11 | 0 | 0 | 0.046 | 1 | 1.000 |  |  |  |  |  |  |  |
|  |  |  |  |  |  |  | 12 | 0 | 0 | 0.012 | 1 | 1.000 |  |  |  |  |  |  |  |
|  |  |  |  |  |  |  | 13 | 0 | 0 | 0.006 | 1 | 1.000 |  |  |  |  |  |  |  |
|  |  |  |  |  |  |  | 14 | 0 | 0 | 0.002 | 1 | 1.000 |  |  |  |  |  |  |  |

***SI Figure 4. Out-degree distribution of the empirical network (black line) vs. simulated networks (box plot)***

***SI Table 7. Out-degree comparison between the empirical network (Obs.) and simulated networks (Min., Mean, Max.) and Monte Carlo (MC) p-values of the comparison***

*1) Financial support layer 2) Material support layer 3) Labor support layer*

|  | Obs. | Min. | Mean | Max. | MC p-value |  |  | Obs. | Min. | Mean | Max. | MC p-value |  |  | Obs. | Min. | Mean | Max. | MC p-value |
| --- | --- | --- | --- | --- | --- | --- | --- | --- | --- | --- | --- | --- | --- | --- | --- | --- | --- | --- | --- |
| 0 | 6 | 1 | 10.110 | 23 | 0.316 |  | 0 | 4 | 0 | 5.990 | 18 | 0.648 |  | 0 | 19 | 7 | 16.412 | 28 | 0.580 |
| 1 | 22 | 6 | 20.536 | 32 | 0.832 |  | 1 | 8 | 4 | 14.130 | 29 | 0.140 |  | 1 | 19 | 14 | 25.478 | 39 | 0.168 |
| 2 | 21 | 11 | 19.874 | 32 | 0.888 |  | 2 | 24 | 8 | 16.810 | 31 | 0.108 |  | 2 | 20 | 8 | 18.564 | 30 | 0.752 |
| 3 | 14 | 4 | 13.650 | 26 | 0.980 |  | 3 | 16 | 4 | 15.250 | 25 | 0.956 |  | 3 | 9 | 2 | 10.136 | 18 | 0.824 |
| 4 | 11 | 1 | 7.914 | 17 | 0.356 |  | 4 | 12 | 2 | 11.334 | 21 | 0.944 |  | 4 | 7 | 0 | 4.560 | 12 | 0.388 |
| 5 | 0 | 0 | 3.694 | 13 | 0.072 |  | 5 | 6 | 1 | 7.222 | 17 | 0.888 |  | 5 | 4 | 0 | 1.828 | 8 | 0.280 |
| 6 | 3 | 0 | 1.490 | 8 | 0.432 |  | 6 | 5 | 0 | 4.070 | 11 | 0.772 |  | 6 | 0 | 0 | 0.716 | 4 | 0.996 |
| 7 | 0 | 0 | 0.510 | 3 | 1.000 |  | 7 | 1 | 0 | 1.846 | 8 | 0.916 |  | 7 | 0 | 0 | 0.212 | 3 | 1.000 |
| 8 | 1 | 0 | 0.172 | 2 | 0.308 |  | 8 | 1 | 0 | 0.838 | 4 | 1.000 |  | 8 | 0 | 0 | 0.066 | 2 | 1.000 |
| 9 | 0 | 0 | 0.040 | 1 | 1.000 |  | 9 | 1 | 0 | 0.320 | 3 | 0.536 |  | 9 | 0 | 0 | 0.024 | 1 | 1.000 |
| 10 | 0 | 0 | 0.0910 | 1 | 1.000 |  | 10 | 0 | 0 | 0.130 | 2 | 1.000 |  | 10 | 0 | 0 | 0.004 | 1 | 1.000 |
|  |  |  |  |  |  |  | 11 | 0 | 0 | 0.042 | 1 | 1.000 |  |  |  |  |  |  |  |
|  |  |  |  |  |  |  | 12 | 0 | 0 | 0.018 | 1 | 1.000 |  |  |  |  |  |  |  |

***SI Figure 5. Geodesic distance distribution of the empirical network (black line) vs. simulated networks (box plot)***

***SI Table 8. Geodesic distance comparison between the empirical network (Obs.) and simulated networks (Min., Mean, Max.) and Monte Carlo (MC) p-values of the comparison***

*1) Financial support layer 2) Material support layer 3) Labor support layer*

|  | Obs. | Min. | Mean | Max. | MC p-value |  |  | Obs. | Min. | Mean | Max. | MC p-value |  |  | Obs. | Min. | Mean | Max. | MC p-value |
| --- | --- | --- | --- | --- | --- | --- | --- | --- | --- | --- | --- | --- | --- | --- | --- | --- | --- | --- | --- |
| 1 | 176 | 109 | 165.706 | 226 | 0.660 |  | 1 | 236 | 152 | 223.850 | 315 | 0.704 |  | 1 | 134 | 82 | 126.958 | 166 | 0.668 |
| 2 | 289 | 127 | 320.908 | 605 | 0.832 |  | 2 | 525 | 247 | 566.336 | 1037 | 0.816 |  | 2 | 165 | 78 | 192.040 | 331 | 0.632 |
| 3 | 383 | 131 | 530.202 | 1208 | 0.464 |  | 3 | 887 | 367 | 1070.198 | 2017 | 0.564 |  | 3 | 203 | 68 | 260.118 | 554 | 0.592 |
| 4 | 423 | 108 | 684.974 | 1422 | 0.304 |  | 4 | 1092 | 474 | 1259.282 | 1802 | 0.532 |  | 4 | 227 | 46 | 304.968 | 716 | 0.652 |
| 5 | 418 | 84 | 673.162 | 1156 | 0.264 |  | 5 | 982 | 469 | 914.836 | 1250 | 0.724 |  | 5 | 222 | 33 | 306.408 | 741 | 0.656 |
| 6 | 390 | 57 | 522.130 | 863 | 0.352 |  | 6 | 751 | 72 | 472.448 | 851 | 0.092 |  | 6 | 196 | 20 | 266.808 | 710 | 0.700 |
| 7 | 287 | 38 | 344.898 | 678 | 0.540 |  | 7 | 469 | 5 | 201.416 | 632 | 0.084 |  | 7 | 145 | 6 | 207.668 | 601 | 0.652 |
| 8 | 169 | 11 | 205.518 | 435 | 0.720 |  | 8 | 236 | 0 | 79.134 | 503 | 0.108 |  | 8 | 81 | 0 | 148.834 | 465 | 0.468 |
| 9 | 104 | 0 | 115.798 | 316 | 0.960 |  | 9 | 102 | 0 | 30.786 | 344 | 0.144 |  | 9 | 37 | 0 | 100.442 | 294 | 0.372 |
| 10 | 67 | 0 | 63.352 | 270 | 0.808 |  | 10 | 39 | 0 | 12.322 | 221 | 0.196 |  | 10 | 12 | 0 | 64.950 | 242 | 0.308 |
| 11 | 28 | 0 | 33.772 | 193 | 0.880 |  | 11 | 12 | 0 | 5.096 | 171 | 0.204 |  | 11 | 2 | 0 | 40.716 | 220 | 0.272 |
| 12 | 10 | 0 | 17.746 | 164 | 0.880 |  | 12 | 2 | 0 | 2.160 | 140 | 0.272 |  | Inf | 4582 | 1836 | 3927.576 | 5624 | 0.500 |
| 13 | 1 | 0 | 9.300 | 138 | 1.000 |  | Inf | 673 | 154 | 1166.652 | 2966 | 0.292 |  |  |  |  |  |  |  |
| Inf | 3261 | 529 | 2309.658 | 5315 | 0.264 |  |  |  |  |  |  |  |  |  |  |  |  |  |  |

***SI Figure 6. Edgewise shared partner distribution of the empirical network (black line) vs. simulated networks (box plot)***

***SI Table 9. Edgewise shared partner comparison between the empirical network (Obs.) and simulated networks (Min., Mean, Max.) and Monte Carlo (MC) p-values of the comparison***

*1) Financial support layer 2) Material support layer 3) Labor support layer*

|  | Obs. | Min. | Mean | Max. | MC p-value |  |  | Obs. | Min. | Mean | Max. | MC p-value |  |  | Obs. | Min. | Mean | Max. | MC p-value |
| --- | --- | --- | --- | --- | --- | --- | --- | --- | --- | --- | --- | --- | --- | --- | --- | --- | --- | --- | --- |
| 0 | 131 | 100 | 139.696 | 188 | 0.604 |  | 0 | 127 | 110 | 146.524 | 180 | 0.112 |  | 0 | 108 | 78 | 105.428 | 135 | 0.812 |
| 1 | 29 | 1 | 24.614 | 58 | 0.632 |  | 1 | 80 | 24 | 70.446 | 135 | 0.648 |  | 1 | 25 | 1 | 21.158 | 54 | 0.588 |
| 2 | 16 | 0 | 1.350 | 14 | 0.000 |  | 2 | 22 | 0 | 6.260 | 23 | 0.012 |  | 2 | 1 | 0 | 0.366 | 4 | 0.524 |
| 3 | 0 | 0 | 0.044 | 3 | 1.000 |  | 3 | 7 | 0 | 0.588 | 5 | 0.000 |  | 3 | 0 | 0 | 0.006 | 1 | 1.000 |
| 4 | 0 | 0 | 0.002 | 1 | 1.000 |  | 4 | 0 | 0 | 0.032 | 2 | 1.000 |  |  |  |  |  |  |  |

**Reference**

1. Marsden, P. V. Network Data and Measurement. *Annu Rev Sociol* **16**, 435–463 (1990).

2. Newman, M. E. J. The mathematics of networks. *The new palgrave encyclopedia of economics* **2**, 1–12 (2008).

3. Borgatti, S. P. Centrality and network flow. *Soc Networks* **27**, 55–71 (2005).

4. Freeman, L. C., Roeder, D. & Mulholland, R. R. Centrality in social networks: ii. experimental results. *Soc Networks* **2**, 119–141 (1979).

5. Kleinberg, J. M., Kumar, R., Raghavan, P., Rajagopalan, S. & Tomkins, A. S. The Web as a Graph: Measurements, Models, and Methods. in 1–17 (1999). doi:10.1007/3-540-48686-0_1.

6. Handcock, M. S., Hunter, D. R., Butts, C. T., Goodreau, S. M. & Morris, M. statnet : Software Tools for the Representation, Visualization, Analysis and Simulation of Network Data. *J Stat Softw* **24**, (2008).

7. Krivitsky, P. N., Koehly, L. M. & Marcum, C. S. Exponential-Family Random Graph Models for Multi-Layer Networks. *Psychometrika* **85**, 630–659 (2020).

8. R Core Team. R: A Language and Environment for Statistical Computing. Preprint at https://www.R-project.org/ (2021).

9. Hunter, D. R., Handcock, M. S., Butts, C. T., Goodreau, S. M. & Morris, M. ergm: A package to fit, simulate and diagnose exponential-family models for networks. *J Stat Softw* **24**, nihpa54860 (2008).

10. Power, E. A. Social support networks and religiosity in rural South India. *Nat Hum Behav* **1**, 57 (2017).

11. Redhead, D., Maliti, E., Andrews, J. B. & Borgerhoff Mulder, M. The interdependence of relational and material wealth inequality in Pemba, Zanzibar. *Philosophical Transactions of the Royal Society B: Biological Sciences* **378**, (2023).

12. Gettler, L. T., Redhead, D., Dzabatou, A. & Lew‐Levy, S. BaYaka forager food sharing networks in the Congo Basin: The roles of gender homophily and kin sharing. *American Journal of Biological Anthropology* **181**, 59–69 (2023).

13. Ready, E. & Power, E. A. Measuring reciprocity: Double sampling, concordance, and network construction. *Network Science* **9**, 387–402 (2021).

14. Hunter, D. R., Goodreau, S. M. & Handcock, M. S. Goodness of Fit of Social Network Models. *J Am Stat Assoc* **103**, 248–258 (2008).

15. Besag, J. & Clifford, P. Sequential Monte Carlo p-Values. *Biometrika* **78**, 301 (1991).

16. Holland, P. W. & Leinhardt, S. Transitivity in Structural Models of Small Groups. *Comparative Group Studies* **2**, 107–124 (1971).
